# Supplementary material for: Financial incentives and deposit contracts to promote HIV retesting in Uganda: A randomized trial
Source: PLoS Med. 2021 May 4;18(5):e1003630. doi: 10.1371/journal.pmed.1003630 (PMC8131095; doi:10.1371/journal.pmed.1003630)
Supplement: S2 Text — (PDF) [file pmed.1003630.s002.pdf]

Innovative Incentive Strategies for Sustainable HIV Testing and Antiretroviral Treatment

A study of the:

Makerere University – University of California, San Francisco (MU-UCSF) Research Collaboration

Sponsored by:
National Institute of Mental Health (NIMH)

Protocol version: 5.0
Version Date: 24 May 2018

Co-Principal Investigator: Gabriel Chamie, MD, MPH
University of California, San Francisco

Co-Principal Investigator: Harsha Thirumurthy, PhD
University of Pennsylvania

Table of Contents

STUDY INVESTIGATORS 4
GLOSSARY OF KEY TERMS 6
1. Introduction 7
1.1 Study Synopsis 7
1.2 Background & Rationale 8
2. Study Objectives 9
3. Study Site & Population 11
3.1 Community Selection 11
3.2 Study Population 11
4. Study Design 11
5. HIV Testing Incentives Trial 13
5.1 Recruitment & Enrollment of Study Participants 13
5.1.1 Recruitment 13
5.1.2 Consent Process 13
5.1.3 Screening for Enrollment 13
5.2 Randomization 13
5.3 Lottery-based Incentive Interventions 14
5.4 Loss Aversion-based Incentives 14
5.5 Fixed Incentive Interventions 14
5.6 Outcomes Measurement 15
5.6.1 HIV Testing 15
5.6.2 Qualitative Measurements 16
5.7 Data & Statistical Considerations 16
5.7.1 Data Collection & Management 16
5.7.2 Analysis 17
5.7.3 Sample Size & Accrual 17
6. HIV Treatment Incentives Trial 18
6.1 Recruitment & Enrollment of Study Participants 18
6.1.1 Recruitment 18
6.1.2 Consent Process 18
6.1.3 Screening for Enrollment 18
6.2 Randomization 18
6.3 Treatment Incentive (Intervention Arm) 18
6.4 Standard of Care - No Incentive (Control Arm) 19
6.5 Outcomes Measurement 19
6.5.1 HIV Virologic Suppression 19
6.5.2 Qualitative Measures 19
6.6 Data Management & Statistical Considerations 19
6.6.1 Data Collection & Management 19
6.6.2 Analysis 20
6.6.3 Sample Size & Accrual 20
7. Repeat HIV Testing Incentives – Pilot Trial 21
7.1 Recruitment & Enrollment of Study Participants 21
7.1.1 Recruitment 21
7.1.2 Consent Process 21
7.1.3 Screening for Enrollment 21
7.2 Randomization 21

7.3 Gain-framed incentive (reward group) 21
7.4 Loss-aversion incentive (deposit contract group) 21
7.5 No incentive (standard of care, information group) 22
7.6 Outcome measures 22
7.6.1 HIV Testing 23
7.6.2 Qualitative interview 23
7.7 Data Management and Statistical Considerations 24
7.7.1 Data Collection & Management 24
7.7.2 Analysis 24
7.7.3 Sample Size & Accrual 24
8. Repeat HIV Testing Incentives Trial 25
8.1 Recruitment & Enrollment of Study Participants 25
8.1.1 Recruitment 25
8.1.2 Consent Process 25
8.1.3 Screening for Enrollment 25
8.2 Randomization 25
8.3 Gain-framed incentive (reward group) 26
8.4 Loss aversion incentive (deposit contract group) 26
8.5 No incentive (standard of care, information group) 26
8.6 Outcome measures 26
8.6.1 HIV Testing 27
8.6.2 Qualitative interview 28
8.7 Data Management and Statistical Considerations 28
8.7.1 Data Collection & Management 28
8.7.2 Analysis 28
8.7.3 Sample Size & Accrual 29
9.0 Sustainability & Cost-Effectiveness 30
9.1 Longitudinal HIV Testing 30
9.2 Cost-Effectiveness Analysis 30
9.3 Qualitative Measures 31
9.4 Analysis 31
10. Human Subjects 32
10.1 Risks to Human Subjects 32
10.2 Adequacy of Protection against risks 32
10.2.1 Informed Consent 32
10.2.2 Maintaining Privacy and Avoiding Stigmatization 32
10.2.3 Minimizing Coercion to Participate in Research 32
10.2.4 Data Security 33
10.2.5 Institutional Review Board Approval 33
10.3 Potential benefits of the proposed research to the participants and others 33
10.4 Importance of the knowledge to be gained 33
11. Publication of Research Findings 34
12. References 34

STUDY INVESTIGATORS

Principal Investigator, UCSF

Gabriel Chamie, MD, MPH
Associate Professor of Medicine
Division of HIV, Infectious Diseases & Global Medicine
San Francisco General Hospital
University of California, San Francisco
UCSF Box 0874, 995 Potrero Avenue
San Francisco, CA 94110, USA
Phone: 415-476-4082, ext. 445
Email: gabriel.chamie@ucsf.edu

Principal Investigator, University of Pennsylvania

Harsha Thirumurthy, PhD
Associate Professor
Department of Medical Ethics and Health Policy
Perelman School of Medicine at the University of Pennsylvania
423 Guardian Drive
Blockley Hall
Philadelphia, PA 19104-4884
Phone: +1-215-898-7136
Email: hthirumu@upenn.edu

Uganda Co-Investigators:

Moses R. Kamya, MBChB, MMed, MPH, PhD
Professor of Medicine
Chair, Department of Medicine
Makerere University
Plot 4B, Kololo Hill Drive
P.O. Box 7972, Kampala, Uganda
Phone: +256-414-533200
Fax: +256-414-540524
Email: mkamya@infocom.co.ug

Dalsone Kwarisiima, MBChB, MPH
Makerere University Joint AIDS Program
Plot 4B Kololo Hill Drive
P.O. Box 7072
Kampala, Uganda
Phone: +256-772-607274
Email: dkwarisiima@mjap.or.ug

United States Co-Investigators

Carol Camlin, PhD, MPH
Associate Professor
Department of Obstetrics, Gynecology and Reproductive Sciences and Center for AIDS Prevention Studies
University of California, San Francisco
50 Beale St., Suite 1200
San Francisco, CA 94105, USA
Phone: (415) 597-4998
Email: Carol.Camlin@ucsf.edu

Diane V. Havlir, MD
Professor of Medicine
Chief, Division of HIV, Infectious Diseases & Global Medicine
University of California, San Francisco
San Francisco General Hospital
995 Potrero Avenue
San Francisco, CA 94110, USA
Phone: 415-476-4082, ext. 424
Email: dhavlir@php.ucsf.edu

James G. Kahn, MD, MPH
Professor of Health Policy and Epidemiology
Philip R. Lee Institute for Health Policy Studies
University of California, San Francisco
UCSF Box 0936
San Francisco, CA 94143-0936, USA
Phone: (415) 476-6642
Email: JimG.Kahn@ucsf.edu

GLOSSARY OF KEY TERMS

**ART**	Antiretroviral therapy
**DALY**	Disability-adjusted life year
**HIV**	Human Immunodeficiency Virus
**HTC**	HIV Testing and Counseling
**MoH**	Ministry of Health
**RCT**	Randomized, controlled trial
**SEARCH**	Sustainable East Africa Research in Community Health
**SSA**	Sub-Saharan Africa
**TasP**	Treatment as Prevention
**VCT**	voluntary counselling and testing

1. Introduction
Despite the investment of considerable resources in HIV testing, over half of adults globally remain unaware of their HIV status. Men in particular fall well below international HIV testing targets [1]. In Uganda's 2011 AIDS Indicator Survey, >50% of 15-49 year old men have never tested for HIV [2]. Whereas women often access testing around childbirth, men access health care (including HIV testing) more rarely and as a consequence are diagnosed later in HIV disease [3, 4]. HIV-infected men are also less likely to link to care than women.[5] Both the male testing and linkage to care "gaps" pose a challenge for the implementation of combination HIV prevention strategies, including treatment as prevention (TasP). Identifying interventions to increase HIV testing among men – particularly those engaged in high-risk behaviors – is thus an HIV prevention priority.

Prior research has shown that economic incentives – the offer of rewards conditional on undertaking a certain action – can be a cost-effective intervention for modifying health behaviors [6, 7]. However, most incentive-based approaches have not utilized key insights from behavioral economics, missing an important opportunity to develop interventions that are more effective *and* less expensive. One insight is that lottery-based incentives may be more effective than small, fixed incentives [8-10]. Importantly, lottery-based incentives may be most appealing to men with risk-seeking preferences, making this intervention particularly well suited for promoting HIV testing among high-risk men. A second insight is that because individuals display "loss aversion" – a strong reluctance to lose something they already own [8, 11] – framing incentives as items to be lost if one does not get tested can be more effective than framing them as items to be won if one gets tested.

Using behavioral economics to develop "smarter" incentive interventions can pave the way for targeted, low-cost ways to identify men who are unaware of their infection and link them to care for prompt initiation of antiretroviral therapy with HIV virologic suppression. This study in Uganda will evaluate the effectiveness and sustainability of innovative incentive strategies to promote HIV testing among men and treatment among HIV-infected adults in rural Uganda. Qualitative research methods will be integrated throughout to elucidate the psychosocial pathways through which incentive strategies affect behaviors.

1.1 Study Synopsis
The success of combination HIV prevention efforts, including HIV treatment as prevention, hinges on universal, routine HIV testing with linkage to care and antiretroviral treatment initiation after HIV diagnosis. The proposed study will evaluate the comparative effectiveness and sustainability of innovative incentive strategies, informed directly by behavioral economics and decision psychology, to promote HIV testing among men and HIV and treatment among HIV-infected adults in rural Uganda.

We will assess the comparative effectiveness of novel interventions designed to increase HIV testing among men who are unaware of their infection in rural Ugandan communities (**Aim 1**). About 3,000 men will be randomized to interventions that encourage them to get HIV tested in their communities. In **Aim 2**, we will study the efficacy of financial incentives for promoting initiation of and facilitating maintenance of antiretroviral therapy (as measured by HIV virologic suppression) among HIV-infected persons identified during a community-based HIV testing campaign. About 400 men and women with HIV will be randomized to receive financial incentives for achieving (or maintaining) an undetectable HIV viral load vs. no incentives (standard of care). In **Aim 3,** we will assess loss aversion strategies for promoting retesting for HIV among high-risk individuals: this aim will first be developed through a pilot study, and then refined for the larger comparative trial. Finally, **Aim 4** will explore the *sustainability* of incentives by examining the effect of one-time incentives on *future* HIV testing behavior. Aim 4 will also assess the cost-effectiveness of incentives, compared with each other. Qualitative research will be integrated into most Aims. Table 1 provides an overview:

Table 1. Overview of studies to assess effectiveness of incentives to increase HIV testing and treatment uptake.

	Study population	Study design and interventions	Primary outcomes
**Aim 1**	About 3,000 men in up to 4 rural Ugandan communities	Randomized trial<br>1. Lottery-based incentives<br>2. Loss aversion incentives<br>3. Fixed incentives<br>In-depth interviews with men from each group who do and do not seek HTC	1. Uptake of HIV testing<br>2. Proportion of men who uptake HIV testing who are HIV-positive

**Aim 2**	About 400 HIV-infected persons who participate in HIV testing in Aim 1 study communities	Randomized trial<br>1. Incentive for HIV virologic suppression, with escalating value for sustaining virologic suppression<br>2. Standard of care (no incentives)	HIV RNA <400 copies/mL six months after randomization
**Aim 3 - Pilot**	About 120 high risk, HIV-negative adults.	Randomized trial<br>1. Loss-framed incentive (with or without endowment) for repeat test<br>2. Gain framed incentive for repeat testing<br>2. No incentive for repeat testing	1. Feasibility and acceptability of loss aversion delivery strategies<br><br>2. Comparative uptake of repeat HIV testing
**Aim 3 - Trial**	About 525 high risk, HIV-negative adults	Randomized trial<br>1. Loss-framed incentive for retesting for HIV<br>2. Gain framed incentive for retesting for HIV<br>2. No incentive for retesting for HIV	1. HIV retesting uptake at both 3 and 6 months following randomization
**Aim 4**	Same as Aim 1 study population	1. Measurement of Aim 1 participants' HIV testing behavior in subsequent years without incentives<br>2. Cost-effectiveness analyses	1. Uptake of HIV testing 1 and 2 years after use of incentives<br>2. Cost per added person testing and linking to care, & per averted DALY

1.2 Background & Rationale

Low awareness of HIV status among men in Africa *despite* increased availability of HIV testing services is a major barrier to implementing combination HIV prevention strategies. The male HIV testing gap is readily apparent in east Africa: in Uganda and Kenya 55% and 37% of men respectively have *never* HIV tested, in contrast to 34% and 20% of women [2, 12]. Men are also less likely to have *recently* tested and more likely to be unaware of their HIV infection [13]. The impact of this can be seen throughout the HIV care cascade, as HIV-infected men are less likely to link to care, enter care with more advanced disease, are less likely to receive antiretroviral therapy (ART), and have higher mortality than HIV-infected women [3, 4, 14-16]. *Given the public health import of HIV testing as the gateway to combination prevention, it is essential to have simple and low-cost interventions to increase HIV testing and treatment among men.*

Economic theories of decision making provide a strong rationale for using small economic incentives to promote healthy behaviors. Incentives can reduce the "price" of a behavior and increase demand for it. Behavioral economics provides an added rationale; with insights from psychology and economics, it points to decision making biases stemming from two types of **time preferences**: a) "present-bias" – the placement of disproportionate weight on *present* vs. *future* costs and benefits [17, 18], and b) "temporal discounting" -- the discounting of future costs and benefits. Thus, even with large benefits to adopting a behavior, individuals may fail to do so if its benefits are delayed and its costs (economic *or* psychological) are immediate. *This implies that individuals can be "nudged" towards healthy behavior with incentives that offer an immediate benefit* [7, 10, 19]. Incentive-based interventions have proven to be acceptable in many settings and found to have minimal risk of coercion. For example, cash and in-kind incentives have modified health behaviors ranging from vaccination uptake, smoking cessation, to HTC [7, 20-24]; they have even been widely used by governments [25, 26]. Our studies in east Africa show that small, fixed incentives are effective in *partially* modifying men's HIV behaviors, such as circumcision and HTC uptake [27, 28]. We have also undertaken qualitative studies to assess the ethics and acceptability of incentives in east Africa [29].

HIV testing and treatment utilization are health behaviors ideally suited for incentive-based interventions. The immediate "costs" of testing, for example, include not just transport costs but also psychosocial ones such as stigma and fear of testing HIV-positive or disclosure [30-33]. The benefits of testing, on the other hand, are more in the future and include the prospect of ART if one tests HIV positive. *Incentives are ideal in this case because they create immediate benefits to testing and even offset some immediate costs. However, the standard approach of offering fixed incentives does not fully achieve desired behaviors and fails to realize the entire potential of incentives by not incorporating ideas from behavioral economics*. For some, small incentives may be overlooked due to the *"peanuts effect"* (rewards may be "small peanuts", not worth the effort) [9], a phenomenon supported by our pilot data [28]. Behavioral economics suggests novel approaches that may be *more effective*, and *less costly*.

Lotteries may be more effective in promoting HIV testing than small, fixed incentives. In seminal research, Kahneman and Tversky's *Prospect Theory* showed that people place a greater decision weight on

probabilities near zero [8]. Thus lotteries with low probabilities of winning large prizes may be more appealing than a fixed incentive that is equal in its *mathematical* expected value. Lottery-based incentives may also be more effective because people tend to pay considerable attention to the *magnitude* of rewards [8, 34]. The *anticipated emotion* of winning a prize can be influential in driving behavior [34]. Lottery-based incentives may be ideal for implementing *targeted testing* and promoting HIV testing among higher risk men. Economists have shown that individuals' **risk preferences** (i.e. attitudes towards risk) are an important predictor of their interest in lotteries [35, 36], with individuals displaying "risk seeking" preferences having the most interest. Such preferences have been associated with health behaviors such as use of preventive medical care and cigarette smoking [37-39]. The men in our study who find lottery-based incentives *most* appealing may be those with risk seeking preferences, a group likely to include HIV-infected persons and those engaged in high risk behaviors.

Loss aversion is a prominent feature of human decision-making behavior that, if properly incorporated into the design of incentive-based interventions, can successfully promote HIV testing. Another central insight from behavioral economics is that individuals display "loss aversion" in their decision making; they have an implicit mental accounting system in which *gaining* something is less motivating than *losing* something of similar value [8, 11, 40]. While loss aversion has been put into practice for some behaviors in high- and middle-income countries by way of "commitment contracts" in which people commit their own money in advance and agree to forgo the money if they fail to undertake a behavior or achieve a target outcome [41], it has not been attempted in low-income countries due to obvious ethical and practical obstacles to imposing penalties on people living in poverty. To overcome these obstacles, we propose a novel loss aversion intervention in which a sense of ownership is generated for an incentive in advance of HIV testing, and participants are informed they will "lose" their reward if they do not HIV test. Our solution relies on the theory that people are sensitive to potential rewards being *framed a*s gains or losses [40, 42, 43]. Just as Rothman, Salovey and others have applied this theory in studies of framing health communication [44, 45], framing incentives as losses can be effective [46]. We will also test the "commitment contract" (or "deposit contract") interventions as these have shown promise in some studies. Assessing whether it is possible to apply the principle of loss aversion and develop interventions that can better motivate individuals to test for HIV is a central motivation of Aim 1 and the Aim 3 pilot.

From the standpoint of sustainability, it is important to learn whether *routine* HIV testing can be encouraged by a *one-time* incentive. Repeat HIV testing is essential for the success of combination prevention [47]. *In the 2012 Kenya AIDS Indicator Survey, for example, >90% of persons who were unaware of their infection reported they were HIV-negative based on their last test result* [12]. There is some evidence that short-term incentives can result in behavior changes that persist after incentives are removed [24]. We will directly address whether the incentives for HTC can increase repeat testing among men in subsequent years within the same communities. Such an effect would enhance the budgetary sustainability of our incentive strategies.

Frequent retesting for HIV infection (more often than annually) is recommended in high-risk individuals,[48] but optimal strategies to promote retesting in sub-Saharan Africa remain unknown. As global efforts continue to reduce HIV incidence through "test and treat" approaches, it is clear that one-time, near-universal HIV testing initiatives will need to be followed by retesting services that offer repeat HIV testing to high-risk individuals living in communities in which universal HIV treatment is available.[49, 50] In addition to promoting HIV testing in persons who have not previously HIV tested, incentives may be effective in promoting longitudinal, repeat HIV testing in people who test HIV antibody negative but remain at high-risk for HIV infection. However, whether incentives are needed to promote repeat HIV testing in high-risk groups, and if so, what type of incentive approach is most likely to be effective remains unknown. We will address these questions in a comparative effectiveness study of incentives that leverage loss aversion vs. traditional gain-framed incentives vs. no incentives, for repeat HIV testing in high-risk individuals who test HIV negative at enrollment.

2. Study Objectives

Aim 1. **Assess the comparative effectiveness of lottery, loss aversion, and fixed incentives for increasing HIV testing among men.** Adult men living in our study communities in rural Uganda will be

randomized to one of three incentive approaches and different incentive amounts that encourage HIV testing. The three incentive approaches will be: 1) Lottery: Men who come for HIV testing will have a chance to instantly win relatively high value prizes (probabilities of winning will range from 1-5%). 2) Loss aversion: Information about incentives for HIV testing will be framed in such a way that it leverages the principle of loss aversion. 3) Fixed: Those who come for HIV testing will receive small fixed prizes. The primary outcome will be HIV testing uptake after randomization. Our hypothesis is that lottery and loss aversion incentives will result in significantly higher testing uptake than fixed incentives. We also hypothesize that the proportion of testers in each arm who are HIV-infected (secondary outcome) will be highest with lottery-based incentives. In sub-samples of men who do and do not test, we will conduct in-depth interviews to assess perceptions, attitudes and preferences related to incentives that may affect how incentives influence testing.

Aim 2. Determine whether HIV-infected men and women are more likely to achieve and maintain HIV virologic suppression if offered financial incentives vs. no incentives (standard of care). Two economic and behavioral factors can serve as barriers to engaging in HIV treatment among HIV-infected persons: (a) present bias - the tendency to place greater weight on immediate costs (i.e. transport costs or missing work) than on delayed benefits (maintaining or restoring health with HIV care and antiretroviral therapy); and (b) the perceived absence of any immediate consequence to delaying health-seeking behavior, including treatment initiation. We hypothesize that a financial incentive will be more effective than no incentive in promoting HIV virologic suppression (a measure of success in ART adherence and navigation of the HIV treatment cascade) because incentives capitalize on present bias by drawing attention to a salient, immediate benefit of initiating and/or maintaining treatment, and leverage loss aversion by generating implicit loss as a result of delaying the decision to initiate ART. By incentivizing adults who have just obtained an HIV-positive result to achieve virologic suppression over the next 6 months, the intervention incentivizes multiple behaviors including prompt linkage to care, initiation of ART, and adherence to ART.

Aim 3 - Pilot. Assess incentives that leverage loss aversion to increase repeat HIV testing among high-risk populations. In order to assess the feasibility of leveraging loss aversion to increase repeat HIV testing, HIV-negative adults who are at high risk of HIV acquisition and have just tested for HIV will be randomized into one of several different incentive strategies that encourage repeat HIV testing. The incentive arms will either: a) leverage loss-aversion by requesting participants to make an initial voluntary deposit that they will lose if they do not test for HIV at a later date; or b) use a standard gain-framed incentive strategy, in which participants are told they will receive an incentive for testing again for HIV at a later date. We will compare these two types of incentive strategies to a no incentive arm as well. Results from this pilot study will be used to inform how best to implement loss aversion-based incentives in a larger trial, and provide preliminary data to guide sample size estimates for a larger trial comparing loss aversion vs. gain-framed incentive-based strategies vs. no incentive, on the outcome of repeat HIV testing. We hypothesize that loss aversion incentives will be feasible (i.e. ≥50% of eligible adults will be willing to participate), and will result in significantly higher testing uptake than either gain-framed incentives or no incentives.

Aim 3 – Trial. Assess the comparative effectiveness of deposit contracts (a form of incentives that leverages loss aversion) vs. game-framed incentives, compared to no incentives (control), to promote repeat HIV testing among high-risk HIV-uninfected adults. In our Aim 3 pilot trial, we assessed the feasibility and acceptability of deposit contracts: a loss aversion-based strategy to incentivize retesting for HIV. As deposit contracts were found to be highly acceptable and feasible in our Aim 3 pilot in August-December 2017 (>90% of participants in the deposit contract group made deposits into the study contracts), we will now proceed with a larger trial of sufficient sample size to compare the effectiveness of loss aversion and gain-framed incentive approaches vs. no incentives, on the outcome of repeat HIV testing. We hypothesize that deposit contracts (loss aversion-based incentives) will result in significantly higher HIV retesting uptake 3- and 6-months after enrollment than either gain-framed incentives or no incentives.

Aim 4. Assess the sustainability of providing incentives for HTC and the cost-effectiveness of novel incentive strategies for HTC and HIV virologic suppression. We will examine the extent to which incentives in one year (Aim 1) affects participation in subsequent testing efforts without incentives. We will also assess effects of the Aim 2 intervention on viral suppression 6 months after incentives are discontinued, i.e. at 12 months. This will reveal whether incentives increase or decrease men's motivation for future HIV testing. We

will also calculate the incremental cost of incentives per additional person tested and enrolled in care as well as the cost per disability-adjusted life year (DALY) averted.

Our overall objective is to generate evidence on how best to use low-cost incentives to promote HIV testing among adult men and maximize the benefits of ART among HIV-infected adults in rural Uganda, and to critically evaluate the sustainability of such an intervention.

3. Study Site & Population

3.1 Community Selection

We will conduct study activities in rural Uganda, enrolling adults (≥18 year old) from up to four communities of 10,000 persons each in southwestern and/or eastern Uganda. The study communities will be selected from the Ugandan candidate communities that met the initial trial eligibility for the ongoing Sustainable East Africa Research in Community Health (SEARCH) HIV test and treat Trial (NCT: 01864603; Co-PIs Chamie and Thirumurthy are Investigators in the SEARCH Trial), *but were not enrolled in the SEARCH trial.* The SEARCH Trial consists of 32 communities selected from 52 candidate communities that met the initial trial eligibility criteria of a rural geopolitical community, with population 10,000, within the catchment area of a PEPFAR-supported HIV clinic in southwestern Uganda, eastern Uganda or western Kenya; 20 candidate communities were not enrolled in SEARCH. All candidate study communities are largely agrarian (>70% of adults in commercial or subsistence farming), with pre-existing ART clinics and free facility-based HIV testing services available locally. Working with the SEARCH Trial team, we have already performed ethnographic mapping, and reviewed national census and epidemiologic data for each candidate community, and have established relationships with the Ministry of Health District Health Officer within each community, facilitating our engagement with these well-characterized communities for the Incentives Trial.

In our Aim 3 trial of retesting for HIV among high-risk HIV-uninfected adults, we will conduct study activities in southwestern Uganda in the urban and peri-urban areas in and around Ibanda and Mbarara Districts, as well as the study communities in which Aims 1-2 were implemented. The urban areas in and around Ibanda and Mbarara Districts include multiple venues at which adults congregate for alcohol consumption and are frequented by groups considered at-risk for HIV infection, including commercial sex workers and long-distance truck drivers.

3.2 Study Population

Adult (≥18 year old) men from up to four candidate study communities will be enumerated and enrolled in the Aim 1 study if they meet eligibility criteria. In Aim 2, men and women who test HIV-positive will be enrolled in the treatment incentives trial. In the Aim 3 Pilot, men and women who are HIV-negative and considered at high-risk of HIV infection will be enrolled in the pilot trial. In the Aim 3 trial (following the Aim 3 Pilot), men and women (≥18 years old and <60 years of age) who are HIV-negative, recruited at high-risk venues in southwestern Uganda, and considered at high-risk of HIV infection will be enrolled. In our Aim 4 activities, we will conduct longitudinal follow-up of the HIV testing behavior of men enrolled in Aim 1.

4. Study Design

a. HIV Testing Incentives Trial (Aim 1): Randomized, controlled trial (RCT) of three types of incentive strategies (lottery-based, loss aversion-based, and fixed incentives) and multiple incentive amounts among adult male residents of rural Ugandan study communities. Eligible men will be randomized in an equal ratio to study groups.

b. HIV Treatment Incentives Trial (Aim 2): Randomized, controlled trial of a financial incentive strategy (with escalating incentive amounts for early treatment initiation and treatment maintenance vs. the no incentive standard of care) on the primary outcome of HIV virologic suppression (plasma HIV RNA <400 copies/mL 6 months after randomization) among HIV-infected adult residents of rural Ugandan study communities. Eligible adults will randomized in a 1:1 ratio.

c. Repeat HIV Testing Incentives (Aim 3 Pilot Trial): Randomized, controlled pilot trial of loss aversion vs. gain-framed incentive strategies vs. no incentive for the primary outcome of acceptability and feasibility of participation in a loss aversion strategy, to promote repeat HIV testing among HIV-negative, high-risk residents of rural Ugandan study communities. Eligible adults will be randomized to a control group, a gain-framed incentive group, or loss-aversion based group in a 1:1:3 ratio.

d. Repeat HIV Testing Incentives (Aim 3 Trial): Randomized, controlled trial of loss aversion (deposit contract) vs. gain-framed incentive strategies vs. no incentive (control) for the primary outcome of retesting for HIV 3- and 6-months following enrollment among HIV-negative, high-risk adults recruited from study sites in southwestern Uganda. Eligible adults will be randomized to the three groups (control, gain-framed and loss aversion incentive groups) in a 1:1:1 ratio.

e. Sustainability and Cost-Effectiveness (Aim 4):

i. *Sustainability of incentives on HIV testing behavior*: Prospective study of HIV testing uptake at community HIV testing venues up to four years after HIV Testing Incentives RCT participants took part in the RCT.
ii. *Cost-effectiveness*: We will measure the full resources and associated costs of implementing the incentive interventions for HIV testing using micro-costing (to quantify resources used and unit costs), and time and motion logs of study staff.

5. HIV Testing Incentives Trial

5.1 Recruitment & Enrollment of Study Participants

5.1.1 Recruitment
Prior to the start of the study, study team members will meet with local officials and community representatives to discuss the study and plans for the enumeration of adult men. Using a map of the boundaries of the selected communities, study staff will systematically cover the entire area within the boundaries to identify and enumerate all households.

5.1.2 Consent Process
An adult household member in each home will be visited during recruitment and will provide oral informed consent before information in collected on several variables about all household members, including the following:

- Name
- Relationship to head of household
- Contact information, including mobile phone number
- Demographic information such as age, gender, occupation and marital status.
- A fingerprint of each household member will be collected with the use of a fingerprint biometric device. (We have found this approach to be highly acceptable and had considerable success with in the ongoing SEARCH Trial in Kenya and Uganda)
- Social networks assessment
- The household's location will be recorded using hand-held GPS receivers.

5.1.3 Screening for Enrollment
Following the census, study staff will review the eligibility criteria among household members and seek to recruit those who meet eligibility criteria (described below). Study staff will review the written informed consent form with eligible individuals and those who provide written consent to study enrollment will have the following performed:

- Baseline questionnaire
- Randomization to incentive strategy (Section 5.2 below)

We will attempt to enroll eligible individuals who are not available during the household visit by returning to the home at times when they are likely to be available.

5.1.3.1 Inclusion Criteria
(a) Male;
(b) Aged ≥18 years;
(c) Reside in one of the selected communities;
(d) Consent to randomization to incentive strategy.

5.1.3.2 Exclusion Criteria
(a) Migration out of study community for ≥6 months per year in the year prior to enrollment.
(b) Intention to move away from the community.

5.2 Randomization
We will compare two theoretically motivated incentive interventions to a standard, fixed incentive intervention. <u>Each intervention will seek to encourage HTC uptake at testing venues in the community (described below)</u>. The incentive interventions will have up to three different values in order to inform researchers and policymakers about the optimal incentive size. The *expected value* of each incentive intervention, within each tier of incentive value, will be the same so that we can draw unambiguous conclusions about the reasons why some incentive interventions work better than others. We will select up to three tiers of incentive value (e.g. low, medium and higher value incentive values), with the specific amounts of expected values per person in US dollars for the incentives determined during pre-enrollment community advisory board meetings. The expected value of the *highest* tier of incentive value will not exceed $10/person, an amount that resembles

what has been used in some other studies in the region [27, 51], and that also corresponds to the sum of transport costs and the opportunity costs of time for some segments of the population.

Study participants will be randomized in equal ratios to the three incentive strategies and to up to three tiers of incentive value, as described below. Randomization will be performed with computer-generated assignments that are grouped in blocks so as to ensure roughly equal sizes in each study group.

5.3 Lottery-based Incentive Interventions

Study staff will inform men that if they come for an HIV test, they will be entered into a lottery that offers a chance to win high-value prizes. Staff will emphasize that *only those who come for HIV testing* will be entered into the lottery and that not everyone will win a prize. Participants will be informed at enrollment about the list of prizes and corresponding probabilities of winning them, in terms that are understandable to men with low numeracy (e.g., "1 in 20" rather than 5%). The probabilities of winning prizes will vary from about 1-5%, with higher value prizes having lower probability. Our pilot data from the SEARCH Trial indicate that lottery-based rewards are feasible and acceptable in communities.

Implementation: After HIV testing and counseling is complete, participants will be given a scratch card or sealed envelope that will reveal if they won a prize (we have used scratch cards in our studies in Kenya previously, and they are also widely used in Uganda). Prizes will be given to participants at the time their scratch card reveals they have won. The exact list of prizes will be determined after further consultation in study communities in year 1.

5.4 Loss Aversion-based Incentives

The prizes (i.e., incentives) given to participants in this arm will be worth approximately the same amount (in US dollars) as the fixed incentive (see below), and the expected value of a lottery prize (see above). At the time of randomization, study staff will either display a specific prize to the participant or will display multiple prizes to the participant and ask him to choose which one he would like to receive if he comes for HIV testing. Participants will be randomized to whether they see a specific prize or multiple prizes. The rationale for the latter strategy is that by actively choosing a prize, participants may form a greater attachment to it and may be more likely to come for HIV testing.

Staff will then spend the duration of the visit generating a sense of familiarity with the prize for the participant. Methods for generating a sense of familiarity will be developed based on experiences theorized to explain the emergence of psychological ownership [52], and after further pilot testing and consultation with community advisory boards in study communities in year 1. Staff will emphasize that men will not receive the prize if they do not participate in HIV testing and that their prize will be returned to them if they get tested. In this way, the incentive is framed as a loss rather than a gain, thereby leveraging loss aversion *while not requiring men in very low-income settings to experience an actual loss* [40, 42].

5.5 Fixed Incentive Interventions

Men will be informed that if they come for HIV testing, they will receive a specific prize (*gain framing*). The prize will be an item (such as a T-shirt) worth the same amount in US dollars as the loss aversion incentive (above), and the expected value of a lottery prize (above). The prize will be described verbally. This gain-framed incentive resembles the form that incentives usually take in most studies and will serve as a comparison to the lottery-based and loss-framed incentives described above.

In all study groups, men will be told about HIV testing venues where incentives will be provided, and encouraged to come for testing due to the various benefits of doing so, which include the availability of free ART, if eligible for ART by country-guidelines, at the local health center.

All participants will also receive text messages on a weekly basis that reminds them about the dates and locations of the HIV testing campaigns to be held in their community. Text messages will mention the incentives for testing that are specific to each participant. A sample of participants in the trial may also be selected at baseline or in subsequent years to low-cost interventions that facilitate planning for when they would like to come for an HIV test. Such planning prompts or "nudges" are intended to increase the likelihood of the desired behavior (HIV testing) at minimal expense without restricting participants' individual autonomy to not go for an HIV test.

5.6 Outcomes Measurement

Primary outcome: **HIV testing uptake.** We will record whether each study participant tested for HIV at a study testing venue (see Section 5.6.1.1 below) within a period that is no longer than 12 months of randomization. We will use electronic fingerprint biometric measurements that link participants in real-time to a study ID at the time of HIV testing to determine whether study participants HIV tested or not (an approach that we have found to be highly acceptable and had considerable success with in the ongoing SEARCH Trial in Kenya and Uganda).

Secondary Outcomes:

a) HIV test result of each participant who gets tested.

b) Risk preferences: We will measure each participant's **risk preferences** at the time of enrollment using a standard approach developed by economists that we have piloted in Kenya previously [35]. Under this approach, a simple questionnaire is used to determine whether participants are risk seeking or risk averse when it comes to financial and non-financial decisions. This in turn can be useful for predicting whether they are likely to seek HTC and be influenced by the provision of certain types of incentives for HTC.

5.6.1 HIV Testing

5.6.1.1 Testing Venues

We will measure HTC at one or more venues within each study community. These may include the community's government-run health facility (where there is likely to be a voluntary counselling and testing [VCT] clinic) or at mobile testing venues established by our study. HTC uptake will be recorded over a period of up to 12 months after enrollment and randomization. Mobile community-based testing venues are an established HTC strategy for increasing male testing, and we have piloted their use in rural Uganda [28, 53]. Other services offered at mobile HIV testing venues may include hypertension and diabetes screening, information and referral for medical male circumcision, instruction on condom use with distribution of free male condoms, men's health counseling, and other Uganda Ministry of Health approved health screening services such as tuberculosis or malaria screening for men with chronic cough or fever, respectively.

At the time they are enrolled in the study, all participants will receive information on when and where to find the testing venues where HTC uptake will be recorded.

5.6.1.2 Testing Procedures

Pre-test counseling: Prior to undergoing diagnostic testing in the field laboratory, pre-test counseling will take place to inform and update participants on the diagnostic services offered and to answer questions.

HIV Testing: Rapid HIV Antibody testing for all participants will take place according to Uganda MoH policy and using the MoH HIV testing algorithm [54]:

1. Initial rapid HIV test
2. All negative results will be informed of their HIV-negative status
3. All positive results will be confirmed with a second rapid HIV test, performed either in series or in parallel with the first test.
4. Discordant results (first test positive, second test negative): participants with discordant rapid tests will undergo a third "tie- breaker" HIV rapid test. Participants with a positive "tie-breaker" test will be informed that they are HIV positive. Participants with a negative "tie-breaker" test will be told that their results are negative.

Non-HIV Health Services. The following services, delivered according to MoH guidelines (and many of which we have implemented in prior studies [28, 53] and the ongoing SEARCH Trial), may be offered at mobile HIV testing venues:

- *Recommended:* Finger-stick blood glucose measure
- *Recommended:* Syphilis screening (RPR)
- *Recommended:* Blood pressure measurement
- *Recommended:* HIV-infected participants: Point-of-care CD4 cell count testing
- *Recommended:* Malaria rapid diagnostic test (RDT) participants with objective fever ($\geq$38.5 degrees Celsius)

- *Recommended:* TB screening (sputum collection for microscopy, *Mycobacterium tuberculosis* culture, or other Uganda MoH approved sputum tests) for men reporting cough for >2 weeks.

Post-test Counseling: All participants, regardless of test results, will receive post-test counseling. Any cases of severe illness identified at a mobile testing venue will be offered transportation to the nearest in-patient health center for hospital-based treatment.

5.6.1.3 Biohazard Containment, Infection Control and Safety

The following procedures and precautions will be instituted throughout the HIV testing activities:

- Universal precautions during blood sticks
- Sharps containers for all lancets used for finger-sticks
- Biohazard trash cans in the van

5.6.2 Qualitative Measurements

In-depth semi-structured interviews: Upon completing measures of HTC uptake, we will recruit men who did and did not HIV test from each study group for in-depth semi-structured interviews in order to better understand: **a)** the *mechanisms* through which the incentives succeeded or failed; and **b)** the *characteristics* of men who do and do not respond to incentives. In accordance with principles of Grounded Theory [55], we will iteratively collect and rapidly analyze interview transcript data, continuing to randomly select participants within theoretically-informed categories (e.g. not only study group but categories such as age group or marital status) until the data attain theoretical saturation. Based on our prior qualitative research in the setting, we anticipate that each study group data will attain saturation with N=10-20 men who opted to test, and N=10-20 men who did not (N=about 100 total). The interview guide will be designed to elicit responses to two thematic areas: i) *men's perceptions, attitudes, and preferences related to the incentives offered*; and ii) *men's other motivations and barriers related to testing*, including qualitative exploration of risk preferences, other psychosocial factors (e.g. fatalism, avoidant coping style, internalized stigma, and perceived masculine gender norms counter to health-seeking behaviors) and contextual factors (e.g. livelihood strategies that interact with responses to incentives and testing). Trained interviewers will pursue lines of questioning following the domains of inquiry outlined above, while allowing participants to discuss salient subjects. Interviews will be audio-recorded, transcribed and translated into English.

5.7 Data & Statistical Considerations

5.7.1 Data Collection & Management

Enumeration and baseline questionnaire data: Staff will collect household enumeration and questionnaire data using hand-held computers (tablets). Before leaving the household, the completed enumeration lists and questionnaires will be checked for mistakes and completeness, ensuring each household has a unique identifier. Data from these devices will be transferred via a secure electronic transfer to our data center facility in Kampala and stored on a secure server.

Household GPS: Each household location will be mapped using a hand-held GPS receiver. Readings will be taken from the door of the household, if possible, or from a point that is most representative of the household. The GPS coordinates for each household will also be recorded in the tablet computer at the time of administering the enumeration questionnaire. GPS data will be synchronized from the GPS to a Microsoft Access database daily and then transferred via a secure electronic transfer to the data center facility in Kampala and stored on a secure server.

Digital fingerprint biometric: A digital biometric identifier based on an electronic fingerprint of each household member will be captured in an electronic database on the hand-held computer and linked to the household member name. A portable fingerprint reader will be connected to the tablet computer via a USB port and the biometric identifier will be saved into the electronic database on the tablet. The database will be transferred via a secure electronic transfer to the data center facility in Kampala and stored on a secure server.

HIV Test Results: Staff working at the HTC venues will record all lab test results, including HIV antibody and CD4 T-cell count results, and HIV diagnosis status information under each participant's study identification

number (ID) in either paper lab logs, or handheld computers. Staff will also record non-HIV screening/test results under each participant's study ID in either paper lab logs or handheld computers. Data from these logs or devices will be transferred via a secure electronic transfer to our data center facility in Kampala and stored on a secure server.

Qualitative Data: Staff will record interviews on audio devices using study IDs (not participant names), and transcribed interviews and translations will be data entered into appropriate software on password-protected study computers. Data from interviews will be transferred via a secure electronic transfer to our data center facility in Kampala and stored on a secure server.

5.7.2 Analysis

Primary analysis: We will compare HTC uptake between the study groups. We hypothesize that HTC uptake among men offered lottery and loss aversion incentives will be higher than uptake among men offered fixed incentives. We will test for significant differences in HTC uptake between the following groups using the 2-tailed χ^2 analysis or other appropriate statistical methods: a) lottery vs. fixed incentive; and b) loss aversion vs. fixed incentive. To further inform policies that use incentives to promote health behaviors, we will compare HTC uptake among those receiving lottery vs. loss aversion incentives. We also hypothesize that those offered larger incentives amounts will have higher HTC uptake.

Secondary analyses: (a) ***HIV-infected men***: We will compare the proportion of HIV testers who are HIV-positive in each of the study groups using 2-tailed χ^2 tests or other appropriate statistical methods. Our hypothesis is that the lottery incentive arm will have a *larger* proportion of HIV-infected men than the other arms, on the basis that it will draw men who are less risk averse.
(b) ***Risk preferences***: We will estimate logistic regression models, or other appropriate statistical models, with an interaction term between study group and the participant's risk preferences. Our hypothesis is that lottery-based incentives will be most effective for men with risk seeking preferences, while other incentives will be most effective for men with risk averse preferences.

Analysis of qualitative data: Translated data will be imported into a qualitative analysis software program, for textual analysis [56] guided by grounded theoretical approaches [55], in which research team members iteratively analyze transcripts and develop a common set of codes describing patterns observed in the data. New codes proposed by research team members will be vetted and defined in relation to existing codes. As new data are collected and reviewed, and data reach analytic saturation, the research team members will independently code the interviews and discuss data segments that are particularly rich or difficult to code. Team members will discuss and resolve divergent coding and interpretations of findings to ensure that codes are applied consistently. Analytical memos will extract and summarize findings following the domains of inquiry outlined above, in order to extract themes and central ideas that explain the meaning of the data.

5.7.3 Sample Size & Accrual

The trial will have about 3,000 total participants. About 1,000 participants will be randomized to each type of incentive strategy (fixed incentives, lottery-based incentives, loss aversion incentives). There will also be equal numbers of participants assigned to each of the incentive amounts that are chosen. The sample size has been chosen to ensure there is adequate power for the primary *and* secondary analyses. For the primary analyses, if we assume HTC uptake is as low as 30% with fixed incentives, the study will have >80% power to detect a difference ($\alpha=0.05$, two-sided) in HTC uptake as small as 6% between the novel incentive and fixed incentive groups. If HTC uptake is 40% with fixed incentives, the study will have >80% power to detect a difference in HTC uptake as small as 7% in each of the novel incentive arms. For secondary analyses, if we assume 40% HTC uptake with fixed incentives (N=400), and 50% with lottery (N=500), we will have >80% power to detect a difference of at least 6% in HIV prevalence between testers in novel and fixed incentive groups (assuming prevalence of 6% in the fixed incentive group).

6. HIV Treatment Incentives Trial

6.1 Recruitment & Enrollment of Study Participants

6.1.1 Recruitment

Men and women who test HIV positive at HIV testing venues within the study communities will be recruited to enroll in the HIV Treatment Incentives Trial. Following post-test counseling at an HIV testing venue, study staff will describe the trial and proceed with obtaining written consent for enrollment from those who meet the eligibility criteria below. Persons who wish to consider enrolling in the trial at a later date will remain eligible for screening for enrollment in the HIV Treatment Incentives Trial, and receive information on how to contact study staff for enrollment at a later date.

6.1.2 Consent Process

Study staff will review the informed consent form with recruited adults who meet the inclusion and exclusion criteria below, and answer any questions about study participation. HIV-infected adults who provide written informed consent and meet criteria for enrollment will proceed to randomization.

6.1.3 Screening for Enrollment

6.1.3.1 Inclusion Criteria

(a) Aged ≥18 years;
(b) Reside in one of the four selected study communities;
(c) Positive result obtained from rapid HIV antibody test algorithm

6.1.3.2 Exclusion Criteria

(a) Intention to move away from the community in the next six months.

Eligible individuals who provide written consent to study enrollment will be administered a brief questionnaire before being randomized.

6.2 Randomization

Eligible participants will be randomized to one of two study arms: an incentive vs. a no incentive (control) arm. Study staff will follow a table of block-randomized numbers to determine which condition (incentive vs. no incentive) each participant receives.

All participants (in both trial arms) will be informed that the local health facility offers ART to all HIV-infected persons and that plasma viral load measurements can take place at either the health facility, their homes, or a convenient location of their choosing. Study staff will provide HIV viral load counseling to all participants, which will include education on what an HIV "viral load" measurement is, and how antiretroviral therapy impacts an HIV-infected person's viral load. In both intervention and control arms, an unconditional cash transfer of up to US$5 will be provided upon enrollment to facilitate linkage to or retention in care. In addition, all participants will be provided with a one-month supply of trimethoprim-sulfamethoxazole and undergo symptom screening for active TB, according to Ugandan Ministry of Health guidelines.

6.3 Treatment Incentive (Intervention Arm)

Participants in the incentive arm will be told that incentive payments will be made based on their study viral load measurements at pre-defined time points (6 weeks, 3 months and 6 months post-randomization). The 6 week incentive will be provided if the participant's plasma HIV RNA level is either <400 copies/mL (if baseline HIV RNA is undetectable), or if the level has had more than a 2 $\log_{10}$ drop compared to the baseline HIV RNA measurement (if baseline HIV RNA is detectable). Incentives at 3 and 6 months will be provided only if a participant's plasma HIV RNA is <400 copies/mL. Study staff will make incentive payments to participants once HIV RNA results that meet the pre-specified cut-off are received. The incentive strategy will be clearly explained verbally and on a printed voucher that is given to them.

The incentives will increase in value when participants are found to meet the pre-specified virologic suppression criteria at 6 weeks, 3 months and 6 months: i) a small, fixed incentive of about US$5 for HIV

virologic suppression within a period of 6 weeks after the HIV test and; ii) an *additional amount* of about US$10 at 3 months, and about $15 at 6 months, if they continue to meet the criteria for virologic suppression (i.e. about US$30 total if HIV virologic suppression is achieved and maintained throughout the 6 months period following randomization). If a participant in the incentive arm does not meet a pre-specified viral load threshold at any time point, no incentive will be given at that timepoint. If the virologic threshold for an incentive is missed at the 6 week or 3 month time-point, the subsequent incentive for an undetectable HIV viral load will be reset to the initial incentive value (up to $5). In this way, the incentive increases in value over time for maintaining virologic suppression – thereby helping maintain the motivation to continue on ART -- and resets to a baseline lower value for missing an HIV virologic threshold.

6.4 Standard of Care - No Incentive (Control Arm)

Participant will be offered standard adherence counseling and an unconditional cash transfer of up to US$5 in order to promote linkage to care.

6.5 Outcomes Measurement

6.5.1 HIV Virologic Suppression

Primary outcome: The proportion of adults with plasma HIV RNA <400 copies/mL six months after randomization.

Secondary outcomes: 1) The proportion of adults with plasma HIV RNA <400 copies/mL 12 months after trial randomization; 2) Mean time to HIV RNA <400 copies/mL; 3) linkage to care within 3 months.

Predictors and mediators: Participants' socio-economic characteristics will be recorded at the time of the HIV test. We will also elicit participants' *time preferences* among those participants who did not participate in the HIV Testing Incentives Trial but who enroll in the Treatment Incentives Trial.

Schedule of measurements: All participants will have HIV RNA measured by phlebotomy (venipuncture) at the following time points: i) enrollment, ii) 6 weeks post-randomization; iii) 12 weeks post-randomization; iv) 24 weeks post-randomization; v) 48 weeks post-randomization. Participants who do not attend a clinic visit for phlebotomy during follow-up time points (6, 12, 24 and 48 weeks post-randomization) will be contacted to meet at a place of their choosing, or tracked in the community if they cannot be contacted by phone.

6.5.2 Qualitative Measures

In-depth semi-structured interviews: We will recruit a random sample of participants who do and do not achieve HIV virologic suppression at 6 months post-randomization from each study arm for in-depth semi-structured interviews. These interviews will seek to understand *mechanisms* through which incentives succeeded or failed, and *characteristics* of adults who did achieve virologic suppression due to the treatment incentive. We will use the same approach to sampling described in the HIV Testing Incentives Trial (Section 5.6.2), and randomly select participants from each of the following groups.

> Incentive group: i) About 10-20 adults who achieved the virologic suppression threshold at 6 weeks; ii) About 10-20 adults who achieve HIV RNA <400 copies/mL at 6 months; iii) About 5-10 adults who have detectable HIV RNA measures at 6 months;
>
> No incentive (control) group: iv) About 10-20 adults who achieve HIV RNA <400 copies/mL at 6 months; and v) About 5-10 adults who have detectable HIV RNA measures at 6 months.

The interview guide will elicit participants' perceptions, attitudes and preferences related to incentives, and psychosocial and contextual factors that influence participants' responses to these incentives and the decision whether or not to take antiretroviral therapy.

6.6 Data Management & Statistical Considerations

6.6.1 Data Collection & Management

Staff will record all HIV RNA measures and study questionnaire data under each participant's study identification number (ID) in either paper lab logs, or password-protected study computers. Data from these logs or devices will be entered and transferred via a secure electronic transfer to our data center facility in Kampala and stored on a secure server.

6.6.2 Analysis

Primary analysis: We will compare the proportion of participants with HIV RNA <400 copies/mL between the two interventions using 2-tailed χ^2 analysis, or other appropriate statistical methods. Our *hypothesis* is that a higher proportion of those offered an incentive will achieve HIV RNA <400 copies/mL than those offered no incentive (the standard of care).

Secondary analysis: Using logistic regression analysis that adjusts for SES, or other appropriate statistical analyses, we will evaluate the impact of *time preferences* (discount rates) on the intervention's effectiveness, using an interaction term between preferences and intervention group. We *hypothesize* that participants with high discount rates (i.e., they discount future benefits heavily and focus on present benefits) are more likely to respond to the incentive than participants with low discount rates.

Qualitative analysis: Data collection and analyses will be similar to those described in Section 5.7.2.

6.6.3 Sample Size & Accrual

Based on data from our pilot studies in Uganda, we anticipate that a minimum of 50% of participants will have HIV RNA <400 copies/mL by six months post-randomization in the control arm. With a sample size of approximately 400 adults, we have >80% power (alpha=0.05, 2-sided) to detect a difference of at least 15% in HIV virologic suppression with the incentive intervention.

7. Repeat HIV Testing Incentives – Pilot Trial

7.1 Recruitment & Enrollment of Study Participants

7.1.1 Recruitment
Prior to the start of the study, study team members will meet with local officials and community representatives to discuss the study and plans for recruiting individuals at higher-risk of HIV acquisition at social venues. In the selected venues (e.g. trading centers, bars, transport stages), study team members will provide information via pamphlets, flyers or postcards, on local HIV testing venues that potential participants may access. All individuals who subsequently come to the HIV testing venues and undergo an HIV test will be given cash or in-kind compensation of up to $10 for their time and transport costs. This compensation will be provided to participants upon completion of the HIV testing.

Men and women who come to the testing venue and test HIV negative will be recruited to enroll in the Repeat HIV Testing Incentives – Pilot Trial. Following post-test counseling, study staff will describe the trial and proceed with obtaining written consent for enrollment from those who meet the eligibility criteria below. Persons who wish to consider enrolling in the trial at a later date will remain eligible for screening for enrollment in the Repeat HIV Testing Incentives – Pilot Trial, and receive information on how to contact study staff for enrollment at a later date.

7.1.2 Consent Process
Study staff will review the informed consent form with recruited adults who meet the inclusion and exclusion criteria below, and answer any questions about study participation. Eligible individuals who provide written consent to study enrollment will be administered a brief questionnaire and fingerprinted before being randomized. Individuals who provide written informed consent and meet criteria for enrollment will proceed to randomization.

7.1.3 Screening for Enrollment

7.1.3.1 Inclusion Criteria
(a) HIV-negative by rapid HIV antibody testing at pilot trial baseline,
(b) Ages 18 – 59 years old,
(c) Attendee of high-risk site of HIV acquisition (e.g. bars, trading centers, etc.) in the region

7.1.3.2 Exclusion Criteria
(a) Intention to move away from the community in the 3 months from time of recruitment.

7.2 Randomization
Eligible participants will be randomized to one of three main arms: 1) a standard gain-framed incentive arm in which participants will be offered an incentive for coming for a repeat HIV test in the future; 2) a loss-aversion incentive in which participants will be asked to voluntarily make a deposit that can be retrieved if they come for an HIV test in the future (i.e. for repeat testing); and 3) a no incentive (control) arm in which participants will simply be encouraged to come for a repeat HIV test in the future. Randomization will occur as follows: study staff will provide participants previously block-randomized scratch cards or envelopes to determine which condition each participant receives. Study staff and participants will not know what study condition an individual is randomized to until the participant scratches the card.

7.3 Gain-framed incentive (reward group)
Study staff will tell participants that they will receive an incentive payment if they come for HIV testing in the near future (within approximately 1-3 months from the time of enrollment). The incentive amount will not exceed US$10. Participants will be encouraged to come for HIV testing in the future and told that they will receive the payment after undergoing the HIV test.

7.4 Loss-aversion incentive (deposit contract group)
In the loss aversion arm, study staff will ask participants if they would like to make a deposit of a certain amount, not exceeding US$10. The amount of compensation given to participants for participating in baseline

HIV testing (as described above in 7.1.1 Recruitment) will facilitate the ability of interested participants to make a deposit. Study staff will retain deposits securely and participants will receive a receipt indicating their deposit amount. Participants will be able to retrieve their deposit when they return for HIV testing in the future. In addition to the deposit, participants will receive interest on their deposit such that the final payment if they return for HIV testing in the future is the same as that in the gain-framed incentive arm. The possibility that participants will lose their deposit if they do not come for an HIV test within specific testing periods (e.g. 1, 3 months) will be communicated clearly by study staff; deposits from individuals who do not test will be retained by the study team and will be used for other study-specific activities. This aspect of deposit contracts is essential in order for the principle of loss aversion to have an impact on participants' HIV testing behavior.

Study staff will also emphasize the voluntary nature of the deposit contract and remind participants that they are not required to make a deposit. Participants who decline to provide a deposit at baseline will still be encouraged to come for an HIV test within a specific testing period (e.g. 1,3 months), but will not receive any incentive for the return HIV test.

We will consider having multiple sub-groups in which participants are asked to make deposits ranging from 25%-50% of the compensation amount provided for baseline testing (see 7.1.1 Recruitment, above); the goal being to learn what amount of deposit is most feasible for participants.

Incentives of this form are sometimes referred to as deposit contracts or commitment contracts, and they apply the principle of loss aversion to help motivate participants who are committed to going for repeat HIV testing to follow through on their commitment. Such types of incentives have previously been implemented in various settings including Kenya, Thailand, and the United States [57-59].

7.5 No incentive (standard of care, information group)

In the no incentive/standard of care arm, study staff will inform participants of the importance of frequent testing for HIV as per Uganda Ministry of Health guidelines for high-risk populations, and to test for HIV at a study-testing facility at a certain interval (e.g. 1, 3 months). The no incentive arm will serve as a comparison to both the loss-framed and gain-framed incentives described above.

7.6 Outcome measures

Primary outcomes:

a) **Feasibility and acceptability of deposits.** We will record the number of eligible adults that agree to continue to participate in the pilot trial by incentive arm, following randomization. We will also record whether a participant randomized in the loss-aversion arms makes a deposit. We will also conduct qualitative interviews to understand why a participant agreed or declined to make a deposit, and the *mechanisms* through which deposits succeeded or failed in promoting repeat HIV testing (see Section 7.6.2).

Secondary outcomes:

a) **HIV testing uptake.** We will record whether each study participant tested for HIV at a study-testing venue (see Section 7.6.1 below) within a period of about 1-3 months after randomization. We will use electronic fingerprint biometric measurements that link participants in real-time to a study ID at the time of HIV testing to determine whether study participants tested for HIV or not. We will compare HIV testing uptake to better inform sample size (power) calculations for planning of a larger trial of loss aversion incentive strategies to promote repeat HIV testing.

b) **HIV test result** of each participant who gets tested.

Predictors and mediators: Participants' demographic and socio-economic characteristics, as well as HIV risk behaviors, will be recorded at the time of the baseline HIV test.

7.6.1 HIV Testing

7.6.1.1 Testing Venues

We will measure HTC at one or more venues within each study community. These may include the community's government-run health facility or at mobile testing sites established by our study (see Section 5.6.1). HTC uptake will be recorded at baseline and over a period of up to 3 months after enrolment and randomization.

At the time they are enrolled in the study, all participants will receive information on when and where to find the testing venues where repeat HTC uptake will be recorded.

7.6.1.2 Testing Procedures

Pre-test counseling: Prior to undergoing diagnostic testing, pre-test counseling will take place to inform and update participants on the diagnostic services offered and to answer questions.

HIV Testing: Rapid HIV Antibody testing for all participants will take place according to Uganda MoH policy and using the MoH HIV testing algorithm [54]:

1. Initial rapid HIV test
2. All negative results will be informed of their HIV-negative status
3. All positive results will be confirmed with a second rapid HIV test, performed either in series or in parallel with the first test.
4. Discordant results (first test positive, second test negative): participants with discordant rapid tests will undergo a third "tie- breaker" HIV rapid test. Participants with a positive "tie-breaker" test will be informed that they are HIV positive. Participants with a negative "tie-breaker" test will be told that their results are negative.

Non-HIV Health Services: The following services, delivered according to MoH guidelines (and many of which we have implemented in prior studies [28, 53] and the ongoing SEARCH Trial), may be offered at mobile HIV testing sites:

- *Recommended:* Finger-stick blood glucose measure
- *Recommended:* Syphilis screening (RPR)
- *Recommended:* Blood pressure measurement
- *Recommended:* HIV-infected participants: HIV RNA ("viral load") and point-of-care CD4 cell count testing
- *Recommended:* Malaria rapid diagnostic test (RDT) participants with objective fever ($\geq$38.5 degrees Celsius)
- *Recommended:* TB screening (sputum collection for microscopy, *Mycobacterium tuberculosis* culture, or other Uganda MoH approved sputum tests) for all individuals.

Post-test Counseling: All participants, regardless of test results, will receive post-test counseling. Individuals who test HIV positive will be immediately offered referral to a clinic that provides ART and counseled as per Uganda MOH guidelines.

Post-test Questionnaire: Participants who come for repeat testing (i.e. 1-3 months after baseline HIV testing) will be asked to participate in a short questionnaire to understand their reasons for testing. Participants who do not come for repeat testing will be contacted and asked to participate in a short questionnaire to understand their reasons for not testing.

7.6.2 Qualitative interview

In-depth semi-structured interviews: We will conduct semi-structured interviews on proportion of study participants in each study arm and subgroup. These interviews will seek to understand the *mechanisms* through which incentives succeeded or failed.

The interview guide will elicit participants' perceptions, attitudes, and preferences related to incentives, including deposits, and psychosocial and contextual factors that influence participants' responses to these incentives and the decision whether or not to test for HIV.

7.7 Data Management and Statistical Considerations

7.7.1 Data Collection & Management

Staff will record all study questionnaire data, HIV test results, and clinic data under each participant's study identification number (ID) in either paper lab logs or password-protected study computers. In-depth semi-structured interviews may be audio recorded. Data from these logs or devices will be entered and transferred via a secure electronic transfer to our data center facility in Kampala and stored on a secure server.

7.7.2 Analysis

Primary analysis:

(a) ***Feasibility and acceptability:*** We will determine the proportion of individuals who agree to make a deposit in the loss aversion-based incentive group, and compare this to the proportion of randomized persons who agree to participate in the gain-framed incentive and no incentive groups following randomization. For qualitative interviews, data collection and analyses will be similar to those described in Section 5.7.2.

Secondary analyses:

(a) ***HIV testing uptake:*** We will compare HTC uptake between the study groups. We hypothesize that HTC uptake among participants offered loss aversion incentives would be higher than uptake among men offered no incentive. We will test for significant differences in HTC uptake between the following groups using the 2-tailed χ^2 analysis or other appropriate statistical methods: a) loss aversion vs. gain-framed incentive; b) loss aversion vs. no incentive; and c) gain-framed vs. no incentive.

(b) ***HIV incidence***: We will compare the proportion of HIV testers who are newly diagnosed as HIV positive in each of the study groups using 2-tailed χ^2 tests or other appropriate statistical methods.

7.7.3 Sample Size & Accrual

The pilot trial will have about 120 total participants, with 25 participants in the no incentive arm, 25 in the gain-framed incentive arm, and about 50-70 participants in the loss-aversion group. The primary goal of the pilot is to gather preliminary data to inform intervention development (feasibility and acceptability) and sample size calculations for a larger trial in the future. In this pilot trial, we will compare the proportion of deposits made in the loss aversion subgroups and HTC uptake among all study groups.

8. Repeat HIV Testing Incentives Trial

8.1 Recruitment & Enrollment of Study Participants

8.1.1 Recruitment

Prior to the start of the study, study team members will meet with local officials and community representatives to discuss the study and plans for recruiting individuals at higher-risk of HIV acquisition at social venues. In the selected venues (e.g. trading centers, bars, transport stages), study team members will provide information via pamphlets, flyers or postcards, on local HIV testing venues that potential participants may access. All individuals who subsequently come to the HIV testing venues and undergo an initial HIV test will be given cash or in-kind compensation of up to $10 for their time and transport costs. This compensation will be provided to participants upon completion of the initial HIV testing.

Men and women who come to the testing venue and test HIV-negative will be recruited to enroll in the Repeat HIV Testing Incentives Trial. Following post-test counseling, study staff will describe the trial and proceed with obtaining written consent for enrollment from those who meet the eligibility criteria below. Persons who wish to consider enrolling in the trial at a later date will remain eligible for screening for enrollment in the Repeat HIV Testing Incentives Trial and receive information on how to contact study staff for enrollment at a later date.

8.1.2 Consent Process

Study staff will review the informed consent form with recruited adults who meet the inclusion and exclusion criteria below and answer any questions about study participation. Eligible individuals who provide written consent to study enrollment will be administered a questionnaire and fingerprinted before being randomized.

8.1.3 Screening for Enrollment

8.1.3.1 Inclusion Criteria

(a) HIV-negative by rapid HIV antibody test at time of recruitment;
(b) Ages 18 – 59 years old;
(c) Reported willingness to retest for HIV in the six months following recruitment;
(d) Sexual risk behavior, defined as at least one of the following self-reported risks:

  i) >1 partner in the 12 months prior to recruitment; *or*
  ii) known HIV-infected sexual partner; *or*
  iii) history of a sexually transmitted infection in the 12 months prior to recruitment; or
  iv) paid or received compensation or gifts for sex in the 12 months prior to recruitment.

8.1.3.2 Exclusion Criteria

(a) Intention to move away from the community for ≥4 consecutive months during the six months following recruitment.
(b) A history of testing for HIV ≥3 times in the 12 months prior to recruitment, by self-report

8.2 Randomization

Eligible participants will be randomized to one of three main arms: 1) a loss-aversion incentive in which participants will be asked to voluntarily make a deposit contract that can be retrieved if they retest for HIV in the future or can be lost if they do not retest; 2) a standard gain-framed incentive arm in which participants will be offered an incentive for retesting for HIV in the future; and 3) a no incentive (control) arm in which participants will be encouraged to retest for HIV in the future, but no incentives will be provided.

Randomization will occur as follows: study staff will provide participants previously block-randomized scratch cards or envelopes to determine which condition each participant receives. Study staff and participants will not know what study condition an individual is randomized to until the participant scratches the card. Eligible adults will be randomized to the three groups in a 1:1:1 ratio.

8.3 Gain-framed incentive (reward group)

Study staff will tell participants that they will receive an incentive payment if they retest for HIV within 3-4 months from the time of enrollment, and a second, equal incentive payment if they retest again (a second time) within 6-7 months from the time of enrollment. Each incentive amount will not exceed US$15. Participants will be encouraged to come for HIV retesting in the future and told that they will receive the payments after undergoing each HIV test.

8.4 Loss aversion incentive (deposit contract group)

In the loss aversion arm, study staff will ask participants if they would like to make a deposit of a certain amount, not exceeding US$15. The amount of compensation given to participants for participating in baseline HIV testing (as described above in 8.1.1 Recruitment) will facilitate the ability of interested participants to make a deposit, as we have learned in the pilot study we conducted in 2017. Study staff will retain deposits securely and participants will receive a receipt indicating their deposit amount. Participants will be able to retrieve their deposit when they return for HIV testing at the 3-month study visit. Participants who attend the 3-month visit and retest HIV negative will be asked if they would like to make a second deposit of a certain amount, not exceeding US$15, to retest a second time at the 6-month study visit. In addition to retrieving their deposit if they return for HIV testing, participants will also receive interest (not exceeding US$5 at each retest visit) on each of their deposits. The possibility that participants will lose their deposit if they do not come for an HIV test within the pre-specified testing periods (3-4 months, and 6-7 months after randomization) will be communicated clearly by study staff; deposits from individuals who do not test will be retained by the study team and will be used for other study-specific activities. This aspect of deposit contracts is essential in order for the principle of loss aversion to have an impact on participants' HIV testing behavior. Our pilot study in 2017 showed that a large majority of individuals were willing to make deposits and that the system of providing receipts worked well.

Adaptive intervention: Following enrollment of 20% of the anticipated number of participants in the loss aversion group, the study investigators will determine the proportion of participants who have agreed to make a baseline deposit. In the event that <15% of loss aversion incentive group participants have agreed to make a deposit, the study investigators may reduce the deposit amount by up to 50% in the loss aversion group for the remaining duration of the Aim 3 trial.

Study staff will emphasize the voluntary nature of the deposit contract and remind participants that they are not required to make a deposit. Participants who decline to provide a deposit at baseline will still be encouraged to come for an HIV test within the pre-specific testing periods (3-4 months, and 6-7 months), but will not receive any incentive for the return HIV test at 3-4 months. Participants who do not make a baseline deposit, but come for retesting at the 3-4 month visit, will have an opportunity to make a deposit at the 3-4 month visit to return for HIV retesting at 6-7 months.

8.5 No incentive (standard of care, information group)

In the no incentive (control) group, study staff will inform participants of the importance of frequent testing for HIV as per Uganda Ministry of Health guidelines for high-risk "key populations",[60] and to test for HIV at a study-testing facility at 3 months and 6 months following randomization. The no incentive arm will serve as a comparison to both the loss aversion and gain-framed incentives described above.

8.6 Outcome measures

Primary outcome:

a) **HIV retesting uptake at 3 and 6 months**. The primary outcome will be defined as the proportion of participants who retest for HIV at a study venue at both 3- and 6-month study visits. Successful retesting uptake will be defined as accessing HIV testing at a study-testing site (see Section 8.6.1 below) within 3-4 months and 6-7 months of randomization. We will use electronic fingerprint biometric measurements that link participants in real-time to a study ID at the time of HIV testing to determine whether study participants tested for HIV or not. Retesting outside of the pre-specified testing "windows" will not be considered as successful HIV retesting uptake.

Secondary outcomes:

- **b) HIV retesting uptake at 3-4 months following randomization.**
- **c) HIV retesting uptake at 6-7 months following randomization.**
- **d) HIV retesting uptake among deposit contract group participants who make deposits**. We will evaluate HIV retesting uptake among participants in the deposit contract group who agree to make a baseline deposit, a 3-4 month deposit, and those who agree to make a deposit at both study baseline and the 3-4 month study visit.
- **e) Cumulative incidence of HIV seroconversion:** We will compare the cumulative incidence of HIV infections in each study group over the 6-7 months of study participation following randomization.

Predictors and mediators: Participants' demographic and socio-economic characteristics, as well as HIV risk behaviors, will be recorded at the time of the baseline HIV test.

8.6.1 HIV Testing

8.6.1.1 Testing Venues

We will measure HIV testing at one or more venues within each study community. These may include the community's government-run health facility or at mobile testing sites established by our study (see Section 5.6.1). HIV testing uptake will be recorded at baseline and over a period of up to 12 months after enrolment and randomization, in order to allow for follow up testing of participants who do not retest within 7 months of randomization.

At the time they are enrolled in the study, all participants will receive information on when and where to find the testing venues where HIV retesting uptake will be recorded.

8.6.1.2 Testing Procedures

Pre-test counseling: Prior to undergoing diagnostic testing, pre-test counseling will take place to inform and update participants on the diagnostic services offered and to answer questions.

HIV Testing: Rapid HIV Antibody testing for all participants will take place according to Uganda MoH policy and using the MoH HIV testing algorithm [54]:

1. Initial rapid HIV test
2. All negative results will be informed of their HIV-negative status
3. All positive results will be confirmed with a second rapid HIV test, performed either in series or in parallel with the first test.
4. Discordant results (first test positive, second test negative): participants with discordant rapid tests will undergo a third "tie- breaker" HIV rapid test. Participants with a positive "tie-breaker" test will be informed that they are HIV positive. Participants with a negative "tie-breaker" test will be told that their results are negative.
5. All participants with two confirmed rapid HIV antibody tests will undergo phlebotomy for HIV viral load measurement in a study laboratory.

Non-HIV Health Services: The following services, delivered according to MoH guidelines (and many of which we have implemented in prior studies [28, 53] and the ongoing SEARCH Trial), may be offered at study HIV testing sites at enrollment and during HIV retesting visits:

- *Recommended:* Finger-stick blood glucose measure
- *Recommended:* Syphilis screening (RPR)
- *Recommended:* Blood pressure measurement
- *Recommended:* HIV-infected participants: HIV RNA ("viral load") and point-of-care CD4 cell count testing
- *Recommended:* Malaria rapid diagnostic test (RDT) participants with objective fever ($\geq$38.5 degrees Celsius)
- *Recommended:* TB screening (sputum collection for microscopy, *Mycobacterium tuberculosis* culture, or other Uganda MoH approved sputum tests) for all individuals.

Post-test Counseling: All participants, regardless of test results, will receive post-test counseling. Individuals who test HIV positive will be immediately offered referral to a clinic that provides ART and counseled as per Uganda MOH guidelines.

Post-test Questionnaire: Participants who come for repeat testing (i.e. 3-4 months and 6-7 months after baseline HIV testing) will be asked to participate in a questionnaire to understand their reasons for retesting. Participants who do not retest for HIV at the 6-month study visit will be contacted, and visited at home or a place of their choosing, and asked to participate in a short questionnaire to understand their reasons for not retesting and to offer follow-up HIV testing, within 12 months of randomization.

8.6.2 Qualitative interview

In-depth semi-structured interviews: We will conduct semi-structured interviews on proportion of study participants in each study arm and subgroup. These interviews will seek to understand the *mechanisms* through which incentives succeeded or failed.

The interview guide will elicit participants' perceptions, attitudes, and preferences related to incentives, including deposits, and psychosocial and contextual factors that influence participants' responses to these incentives and the decision whether or not to retest for HIV.

8.7 Data Management and Statistical Considerations

8.7.1 Data Collection & Management

Staff will record all study questionnaire data, participant digital fingerprint biometric measurements, HIV test results, and clinic data under each participant's study identification number (ID) in either paper lab logs or password-protected study computers. In-depth semi-structured interviews may be audio recorded. Data from these logs or devices will be entered and transferred via a secure electronic transfer to our data center facility in Kampala and stored on a secure server.

8.7.2 Analysis

Primary analysis:

(a) ***HIV retesting uptake:*** We will compare HIV retesting uptake (defined as the proportion of participants retesting for HIV at both 3- and 6-month study visits) between the study groups. We hypothesize that HIV retesting uptake among participants offered loss aversion incentives will be higher than uptake among men offered no incentive, and higher than uptake among men offered gain-framed incentives. We will test for significant differences in HIV retesting uptake between the following groups using the 2-tailed χ^2 analysis or other appropriate statistical methods: a) loss aversion vs. gain-framed incentive; b) loss aversion vs. no incentive; and c) gain-framed vs. no incentive.

Secondary analyses:

(b) ***HIV re-testing uptake at 3-months:*** We will compare the proportion of participants who retest for HIV at the 3-month study visit between the study groups. We will test for significant differences in HIV retesting uptake between the following groups using the 2-tailed χ^2 analysis or other appropriate statistical methods: a) loss aversion vs. gain-framed incentive; b) loss aversion vs. no incentive; and c) gain-framed vs. no incentive.

(c) ***HIV re-testing uptake at 6-months:*** We will compare the proportion of participants who retest for HIV at the 6-month study visit between the study groups. We will test for significant differences in HIV retesting uptake between the following groups using the 2-tailed χ^2 analysis or other appropriate statistical methods: a) loss aversion vs. gain-framed incentive; b) loss aversion vs. no incentive; and c) gain-framed vs. no incentive.

(d) **HIV retesting uptake among deposit contract group participants who make deposits**. We will compare HIV retesting uptake as described for the Aim 3 primary analysis (above), but restrict the deposit contract group participants to the subset who make deposits at baseline and at the 3-4 month study visit. Since this subgroup is likely to be self-selected on the basis of their desire to come for retesting, we will determine the causal effect of the deposit contract on the likelihood of coming for

retesting using instrumental variables analyses in which randomization to the deposit contract group serves as an instrumental variable for a participant's decision to make a deposit.

(e) ***Cumulative HIV incidence***: We will compare the proportion of participants who test HIV antibody positive within 7 months of participant randomization in each of the study groups using 2-tailed χ^2 tests or other appropriate statistical methods. We hypothesize that the proportion of newly diagnosed HIV antibody positive participants will be higher in the control group, due to failure to participate in retesting for HIV.

8.7.3 Sample Size & Accrual

We anticipate that about 30% of participants in the control group will come for retesting at both 3 and 6 months. With a sample size of 525 participants randomized 1:1:1 to the 3 study arms, we will have >80% power (alpha=0.05, 2-sided) to show a difference of at least 15% in retesting rates in each of the intervention groups compared to the control group. With this sample size there will be >90% power to show a difference of at least 18% between the intervention and control groups.

9.0 Sustainability & Cost-Effectiveness

Repeat HIV testing among high-risk individuals is essential for the success of combination HIV prevention. By engaging men in first-time testing as part of our HIV testing incentives trial (Section 5), there may be an important opportunity for establishing healthy behaviors. To examine issues related to the sustainability of offering incentives and the cost-effectiveness of such an approach, we will quantitatively and qualitatively assess *repeat* testing behavior among participants in the study communities where we conduct the incentives trial. We will thereby address an important question that has rarely been examined: do one-time incentives strengthen or weaken individuals' likelihood of testing routinely once incentives are no longer offered? We will also address a central policy issue: the cost-effectiveness of using incentives to promote HTC and HIV treatment.

9.1 Longitudinal HIV Testing

In each of the study communities where the incentive trial was conducted, we will measure HTC uptake at multiple time periods after the start of the incentives trial. The time periods during which we will measure HTC uptake will range from about 12-36 months after the start of the trial so that we can monitor repeat testing period longitudinally. HTC uptake will be recorded at similar HTC venues as those used at baseline, in the incentives trial. All participants will once again receive information on when and where to find the testing venues. In these subsequent time periods, incentives offered for HTC will either be significantly smaller or non-existent, since the objective will be test the durability and sustainability of the effect of offering incentives for HTC initially. As in the incentives trial conducted at baseline, we will use electronic fingerprint biometric measurements that link participants in real-time to a study ID at the time of HIV testing to determine whether study participants HIV tested or not.

9.2 Cost-Effectiveness Analysis

Intervention costs: We will measure the full resources and associated costs of implementing the incentive interventions. Costs will include not just the incentive amounts but also costs required to deliver each intervention (home visits, staff time, prize procurement, supplies, and monitoring). Data will come from two methods: (a) Micro-costing will quantify resources used and unit costs. We will extract data from project expenditure and management records, including purchase logs and human resource records. We will record this information in structured costing spreadsheets that record each resource (e.g., counselor), category (e.g., personnel, supplies), quantity (e.g., hours), and unit costs. (b) Time and motion logs will record how staff divide time among multiple roles (within and outside the incentive intervention) to allow us to reliably apportion effort to incentive-related activities, especially when staff have other roles.

Intervention effects: To measure effectiveness as part of an assessment of the cost-effectiveness of incentives vs. no incentives, we will also use data from four other SEARCH Trial communities to quantify testing and virologic suppression rates for men who did not test at baseline and received no incentives for testing.

We will conduct five linked analyses. In each we will compare among incentive arms and to non-incentive SEARCH communities. (1) *Increased short-term testing:* cost per added person tested and HIV-infected person identified. (2) *Increased HIV virologic suppression:* cost per added person with virologic suppression (i.e., tested and on treatment). (3) *Reduced disease burden:* cost per averted DALY based on improved testing and treatment. This will include modeled lifetime ART health benefits and costs. (4) *Repetition of analyses 1-3 incorporating the long-term (3 year) effects of incentives* on testing (as assessed in this 3rd Aim). If the proportion of participants testing at future HTC campaigns remains similar to the proportion who tested when incentives were offered, the increased yield of testing and HIV-infected individuals identified in those future years – with **no** added incentive cost – will result in more favorable cost-effectiveness ratios. (5) *Cost implications of incentives given a community target testing level.* Increased HTC uptake at mobile venues due to incentives (and thus decreased need for resource-intensive tracking and testing) could generate overall cost *reductions* for identifying persons unaware of their infection. The main outcome metric will be how many additional high-risk men can be tested by spending fixed resources on novel rather than standard incentives.

These analyses will be conducted using customized spreadsheet-based decision tree and clinical progression simulation modeling methods adapted from our past projects [61-63]. Extensive 1-way, multi-way, threshold, and scenario sensitivity analyses will quantify the effects of input uncertainty on results. Our analytic

perspective will be the health system, for direct medical costs, discounting future costs and DALYs at 3% per year.

9.3 Qualitative Measures

In the subsequent years when HTC services will be offered in study communities and HTC uptake will be measured, in-depth semi-structured interviews will be conducted in a random sample of HIV-negative men who tested initially (at baseline, when incentives were offered), and then either continued to test in subsequent years (N = about 10-20) or did not test (N = about 10-20). Interviews will assess the long-term influence of incentives on men's attitudes towards HIV testing. Procedures followed in the in-depth interviews will be similar to those used at baseline, in the incentives trial.

9.4 Analysis

Long-term effects of incentives: We will examine trends in HTC uptake over a period of up to 36 months for the individuals who participate in the incentives trial. To control for time trends in HTC uptake that may bias our results, we will also utilize longitudinal data on HTC uptake by men in the SEARCH Trial communities. We will assess whether HTC uptake is maintained at baseline levels or if instead it declines over time at rates that exceed what is observed in the SEARCH Trial among men who test at baseline.

Cost-effectiveness: We will conduct five linked analyses. In each we will compare among incentive arms and to non-incentive SEARCH communities. (1) Increased short-term testing: cost per added person tested and HIV-infected person identified. (2) Increased HIV treatment: cost per added person with HIV RNA suppression (i.e., tested and achieved or maintained vriologic suppression). (3) Reduced disease burden: cost per averted disability-adjusted life year (DALY) based on improved testing and treatment (with higher and earlier initiation of ART). This will include modeled lifetime ART health benefits and costs. (4) Repetition of analyses 1-3 incorporating the long-term (3 year) effects of incentives on testing (as assessed in this 3rd Aim). If the proportion of participants testing at future HTC campaigns remains similar to the proportion who tested when incentives were offered, the increased yield of testing and HIV-infected individuals identified in those future years – with no added incentive cost – will result in more favorable cost-effectiveness ratios. (5) Cost implications of incentives given a community target testing level. Increased HTC uptake at mobile venues due to incentives (and thus decreased need for resource-intensive tracking and testing) could generate overall cost reductions for identifying persons unaware of their infection. The main outcome metric will be how many additional high-risk men can be tested by spending fixed resources on novel rather than standard incentives. These analyses will be conducted using customized spreadsheet-based decision tree and clinical progression simulation modeling methods adapted from our past projects. Extensive 1-way, multi-way, threshold, and scenario sensitivity analyses will quantify the effects of input uncertainty on results. Our analytic perspective will be the health system, for direct medical costs, discounting future costs and DALYs at 3% per year.

10. Human Subjects

10.1 Risks to Human Subjects

There are relatively few risks to study participants. The primary risks involved in study participation are inadvertent HIV disclosure with associated stigma and inadvertent coercion with incentives to participate in research. Given the sensitive and private nature of the HIV test results to be collected during implementation of the research aims, extra cautions will be put in place to ensure maintenance of privacy, confidentiality, and security of the data obtained. Data collection and storage procedures will include the use of encrypted, password-protected devices and servers.

Another risk stems from the use of incentives to promote HIV testing and treatment. However, the value of the fixed incentives as well as the expected value of the lottery-based incentives are low compared to certain costs of HIV testing such as transportation and opportunity costs. This means that most participants are unlikely to experience a significant economic gain as a result of getting tested or treated for HIV, thereby minimizing the risk of coercion. A recent publication by the Ethics Working Group of the HIV Prevention Trials Network has also made the case that the use of incentives for health promotion does not necessarily undermine individual autonomy [64]. Instead, the incentives can help overcome economic obstacles or motivational deficiencies; they can promote engagement in health-related behaviors that participants regard as beneficial or worthwhile, but do not undertake due to behavioral biases such as present-biased preferences. Our past experience implementing small economic incentive interventions in Kenya and Uganda has also demonstrated that such interventions are acceptable to both communities and ethical review boards [28, 65].

10.2 Adequacy of Protection against risks

10.2.1 Informed Consent

Written informed consent will be obtained from all participants by trained study staff. The study staff will find a private place in or near the home environment; carefully explain the nature and purpose of the study to each potential participant, as well as the various incentive-based interventions they may receive. The consent process will be offered in English as well as the major local dialects in southwestern Uganda (Runyankole) and eastern Uganda (Jopadhola, Iteso, Swahili and Luganda). Our study team has prior experience in obtaining informed consent for research and clinical trials within the cultural context of Uganda. The informed consent procedure has been designed to maximize understanding of potential risks. Participants may decline to participate at any point.

10.2.2 Maintaining Privacy and Avoiding Stigmatization

Study visits to households to recruit, screen, consent and enroll participants will occur in unmarked vehicles in order to reduce the unlikely possibility of stigmatizing study participants. Every effort will be made to ensure privacy is maintained during HIV testing (Aims 1 and 3) and during HIV viral load measurements (Aims 2 and 3). HIV testing will be *confidential* and will be linked to participants by a unique study identification number. Aim 2 HIV viral load measurements (by phlebotomy) will be made at a location of each participant's choosing, and may include home visits, visits to another convenient community location, or clinic-based visits.

10.2.3 Minimizing Coercion to Participate in Research

To minimize the likelihood of persons feeling pressured to participate in research we will emphasize the concepts of individual voluntary choice to participate in research and the need for individuals to respect the voluntary choice of others during the process of obtaining informed consent. Study participants will also be informed that study enrollment can be stopped at any point during the study at their request. It will be explained to participants that incentive payments will be conditional on HIV testing (Aim 1, Aim 3 Pilot, and Aim 3 trial) or a plasma HIV RNA measurement <400 copies/mL (Aim 2), and that participants will be receiving different types incentives as well as different amounts. The risk of coercion is likely to be minimal in this case because the incentives are not so large in value that they substantially exceed the likely costs associated with HIV testing or treatment. Incentives of similar value have been used in a number of different contexts in resource-limited settings to achieve changes in behaviors [27, 51, 66], and we have previously received IRB approval for the use of such amounts.

10.2.4 Data Security
All information will be recorded using study identification numbers, rather than participant names, and stored securely in locked offices at the MU-UCSF data center. All study computers are password encrypted and kept in locked offices.

10.2.5 Institutional Review Board Approval
Approval from the UCSF institutional review board, and Ugandan institutional review boards (the School of Medicine Research and Ethics Committee [SOMREC], and the Uganda National Council for Science and Technology [UNCST]) will be obtained prior to initiation of study activities. All study staff are required to undergo training in human subjects research, and good clinical practice (GCP).

10.3 Potential benefits of the proposed research to the participants and others
The primary benefits of the proposed research to participants are community-based access to free mobile HIV testing along with other men’s health services (such as male condoms), post-test counseling and referral to HIV care if HIV-infected, and receiving the economic incentives for HIV testing, HIV retesting, and effective treatment in Aims 1, 3 and 2, respectively. The benefits for the community may include increased engagement in HIV prevention by men, increased HIV testing among high-risk adults, and increased population-level HIV virologic suppression.

10.4 Importance of the knowledge to be gained
The minimal risk in this study is far outweighed by the importance of the knowledge to be gained. Given the public health importance of achieving higher uptake of HIV testing among men, higher rates of retesting among high-risk adults, and prompt initiation of antiretroviral treatment, it is vital to have a set of proven low-cost interventions that can be used to achieve these objectives. Our study will provide information on the effectiveness of incentives as a strategy that can be deployed in resource-limited settings.

11. Publication of Research Findings

The findings from this study may be published in a medical journal. No individual identities will be used in any reports or publications resulting from the study. The researchers will publish results of the study in accordance with NIMH, UCSF, UNCST, and Makerere University guidelines.

12. References

1. Staveteig, S., et al., *Demographic Patterns of HIV Testing Uptake in Sub-Saharan Africa. DHS Comparative Reports No. 30. Calverton, Maryland, USA: ICF International.* 2013.
2. *Uganda Ministry of Health and ICF International. 2011 Uganda AIDS Indicator Survey: Key Findings. Calverton, Maryland, USA: MOH and ICF International.* 2012.
3. Muula, A.S., et al., *Gender distribution of adult patients on highly active antiretroviral therapy (HAART) in Southern Africa: a systematic review.* BMC Public Health, 2007. **7**: p. 63.
4. Braitstein, P., et al., *Gender and the use of antiretroviral treatment in resource-constrained settings: findings from a multicenter collaboration.* Journal of women's health, 2008. **17**(1): p. 47-55.
5. Govindasamy, D., N. Ford, and K. Kranzer, *Risk factors, barriers and facilitators for linkage to antiretroviral therapy care: a systematic review.* AIDS, 2012. **26**(16): p. 2059-67.
6. Sindelar, J.L., *Paying for performance: the power of incentives over habits.* Health economics, 2008. **17**(4): p. 449-51.
7. Volpp, K.G., et al., *P4P4P: an agenda for research on pay-for-performance for patients.* Health Affairs, 2009. **28**(1): p. 206-14.
8. Kahneman, D. and A. Tversky, *Prospect theory: an analysis of decision under risk.* Econometrica, 1979. **47**(2): p. 263-291.
9. Weber, B.J. and G.B. Chapman, *Playing for peanuts: why is risk seeking more common for low-stakes gambles?* . Organ Behav Hum Decis Process, 2005. **91**: p. 31-46.
10. Loewenstein, G., T. Brennan, and K.G. Volpp, *Asymmetric paternalism to improve health behaviors.* JAMA, 2007. **298**(20): p. 2415-7.
11. Thaler, R., *Toward a positive theory of consumer choice.* Journal of Economic Behavior & Organization, 1980. **1**(1): p. 39-60.
12. *National AIDS and STI Control Programme, Kenya AIDS Indicator Survey 2012: Preliminary Report. Nairobi, Kenya.* 2012.
13. Ng'ang'a, A., et al., *The status of HIV testing and counseling in Kenya: results from a nationally representative population-based survey.* J Acquir Immune Defic Syndr, 2014. **66 Suppl 1**: p. S27-36.
14. Greig, A., et al., *Gender and AIDS: time to act.* AIDS, 2008. **22 Suppl 2**: p. S35-43.
15. UNAIDS, *Global report: UNAIDS report on the global AIDS epidemic 2013.* 2013, UNAIDS: Geneva.
16. Mills, E.J., N. Ford, and P. Mugyenyi, *Expanding HIV care in Africa: making men matter.* Lancet, 2009. **374**(9686): p. 275-6.
17. Laibson, D., *Golden Eggs and Hyperbolic Discounting.* Quarterly Journal of Economics., 1997(112): p. 2.
18. O'Donoghue, T. and M. Rabin, *Doing it now or later.* American Economic Review, 1999. **89**(1): p. 103-124.
19. Thaler, R.H. and C.R. Sunstein, *Nudge : improving decisions about health, wealth, and happiness*. Rev. and expanded ed. 2009, New York: Penguin Books. viii, 312 p.
20. Banerjee, A.V., et al., *Improving immunisation coverage in rural India: clustered randomised controlled evaluation of immunisation campaigns with and without incentives.* BMJ, 2010. **340**: p. c2220.
21. Lussier, J.P., et al., *A meta-analysis of voucher-based reinforcement therapy for substance use disorders.* Addiction, 2006. **101**(2): p. 192-203.
22. Ashraf, N., J. Berry, and J.M. Shapiro, *Can Higher Prices Stimulate Product Use? Evidence from a Field Experiment in Zambia.* American Economic Review, 2010. **100**(5): p. 2383-2413.

23. Fernald, L.C., P.J. Gertler, and L.M. Neufeld, *10-year effect of Oportunidades, Mexico's conditional cash transfer programme, on child growth, cognition, language, and behaviour: a longitudinal follow-up study.* Lancet, 2009. **374**(9706): p. 1997-2005.
24. Charness, G. and U. Gneezy, *Incentives to exercise.* Econometrica, 2009. **77**(3): p. 909-931.
25. Schultz, T.P., *School Subsidies for the Poor: Evaluating the Mexican Progresa Poverty Program.* Journal of Development Economics, 2004. **74**(1): p. 199-250.
26. Lagarde, M., A. Haines, and N. Palmer, *The impact of conditional cash transfers on health outcomes and use of health services in low and middle income countries.* Cochrane database of systematic reviews, 2009(4): p. CD008137.
27. Thirumurthy, H., et al., *Effect of Providing Conditional Economic Compensation on Uptake of Voluntary Medical Male Circumcision in Kenya.* JAMA : the journal of the American Medical Association, 2014. **312**(7): p. 703-711.
28. Chamie, G., et al., *Uptake of Community-Based HIV Testing during a Multi-Disease Health Campaign in Rural Uganda.* PLoS ONE, 2014. **9**(1): p. e84317.
29. Evens, E., et al., *Conditional economic compensation to increase demand for voluntary medical male circumcision: qualitative interviews with male participants in a randomized controlled trial and their partners in Kenya.* 2014.
30. Ostermann, J., et al., *Who tests, who doesn't, and why? Uptake of mobile HIV counseling and testing in the Kilimanjaro Region of Tanzania.* PLoS ONE, 2011. **6**(1): p. e16488.
31. Morin, S.F., et al., *Removing barriers to knowing HIV status: same-day mobile HIV testing in Zimbabwe.* Journal of acquired immune deficiency syndromes, 2006. **41**(2): p. 218-24.
32. Bwambale, F.M., et al., *Voluntary HIV counselling and testing among men in rural western Uganda: implications for HIV prevention.* BMC Public Health, 2008. **8**: p. 263.
33. Kranzer, K., et al., *Individual, household and community factors associated with HIV test refusal in rural Malawi.* Tropical medicine & international health : TM & IH, 2008. **13**(11): p. 1341-50.
34. Loewenstein, G., et al., *Risk as Feelings.* Psychological bulletin, 2001. **127**(2): p. 267-286.
35. Eckel, C.C. and P.J. Grossman, *Forecasting risk attitudes: An experimental study using actual and forecast gamble choices.* Journal of Economic Behavior & Organization, 2008. **68**: p. 1-17.
36. Starmer, C., *Developments in Non-Expected Utility Theory: The Hunt for a Descriptive Theory of Choice under Risk.* Journal of Economic Literature, 2008. **38**(2): p. 332-382.
37. Anderson, L.R. and J.M. Mellor, *Predicting health behaviors with an experimental measure of risk preference.* Journal of health economics, 2008. **27**(5): p. 1260-74.
38. Lahiri, K. and J.G. Song, *The effect of smoking on health using a sequential self-selection model.* Health economics, 2000. **9**(6): p. 491-511.
39. Barsky, R.B., K. Miles, and M. Shapiro, *Preference parameters and behavioral heterogeneity: an experimental approach in the health and retirement study.* Quarterly Journal of Economics., 1997. **112**(2): p. 537-579.
40. Thaler, R.H., *Mental accounting and consumer choice.* Marketing Science, 1985. **4**: p. 199-214.
41. Gine, X., D. Karlan, and J. Zinman, *Put Your Money Where Your Butt Is: A Commitment Contract for Smoking Cessation.* American Economic Journal: Applied Economics, 2010. **2**(4): p. 213-35.
42. Kahneman, D., J.L. Knetsch, and R.H. Thaler, *Anomalies: The Endowment Effect, Loss Aversion, and Status Quo Bias.* Journal of Economic Perspectives,, 1991. **5**(1): p. 193-206.
43. Tversky, A. and D. Kahneman, *The framing of decisions and the psychology of choice.* Science, 1981. **211**(4481): p. 453-8.
44. Rothman, A. and P. Salovey, *Shaping perceptions to motivate healthy behavior: the role of message framing.* Physcological Bulletin, 1997. **121**(1): p. 3-19.
45. Richardson, J.L., et al., *Effect of brief safer-sex counseling by medical providers to HIV-1 seropositive patients: a multi-clinic assessment.* Aids, 2004. **18**(8): p. 1179-86.
46. Bertrand, M., et al., *What's Advertising Content Worth? Evidence from a Consumer Credit Marketing Field Experiment.* Quarterly Journal of Economics., 2010. **125**(1): p. 263-305.

47. Granich, R.M., et al., *Universal voluntary HIV testing with immediate antiretroviral therapy as a strategy for elimination of HIV transmission: a mathematical model.* Lancet, 2009. **373**(9657): p. 48-57.
48. *World Health Organization (WHO) Consolidated Guidelines on HIV Testing Services*. 2015: Geneva, Switzerland.
49. Grabbe, K.L., et al., *Re-Testing and Seroconversion Among HIV Testing and Counseling Clients in Lesotho.* AIDS Educ Prev, 2015. **27**(4): p. 350-61.
50. Johnson, C.C. and E.L. Corbett, *HIV self-testing to scale up couples and partner testing.* Lancet HIV, 2016. **3**(6): p. e243-4.
51. Baird, S.J., et al., *Effect of a cash transfer programme for schooling on prevalence of HIV and herpes simplex type 2 in Malawi: a cluster randomised trial.* Lancet, 2012. **379**(9823): p. 1320-9.
52. Pierce, J.L., T. Kostova, and K.T. Dirks, *The state of psychological ownership: Integrating and extending a century of research.* Review of General Psychology, 2003. **7**(1): p. 84-107.
53. Chamie, G., et al., *Leveraging rapid community-based HIV testing campaigns for non-communicable diseases in rural Uganda.* PLoS One, 2012. **7**(8): p. e43400.
54. *Uganda. Ministry of Health. 2005. Uganda national policy guidelines for HIV voluntary counselling and testing. Kampala: Ministry of Health.*
55. Charmaz, K., *Constructing Grounded Theory*. 2006, Rohnert Park: Sage.
56. Denzin, N.K. and Y.S. Lincoln, eds. *Collecting and Interpreting Qualitative Materials*. 3rd ed. 2008, Sage Publications: Thousand Oaks, CA.
57. Dupas, P. and J. Robinson, *Why don't the poor save more? Evidence from health savings experiments.* American Economic Review, 2013. **103**(4): p. 1138-1171.
58. White, J.S., W.H. Dow, and S. Rungruanghiranya, *Commitment Contracts and Team Incentives: A Randomized Controlled Trial for Smoking Cessation in Thailand.* American Journal of Preventive Medicine, 2013. **45**(5): p. 533-542.
59. Halpern, S.D., et al., *Randomized Trial of Four Financial-Incentive Programs for Smoking Cessation.* New England Journal of Medicine, 2015. **372**: p. 2108-2117.
60. *Uganda Ministry of Health: Consolidated Guidelines for Prevention and Treatment of HIV in Uganda*. 2016.
61. Marseille, E., et al., *The cost effectiveness of home-based provision of antiretroviral therapy in rural Uganda.* Applied health economics and health policy, 2009. **7**(4): p. 229-43.
62. Marseille, E., et al., *Taking ART to scale: determinants of the cost and cost-effectiveness of antiretroviral therapy in 45 clinical sites in Zambia.* PLoS ONE, 2012. **7**(12): p. e51993.
63. Kahn, J.G., et al., *CD4 cell count and viral load monitoring in patients undergoing antiretroviral therapy in Uganda: cost effectiveness study.* Bmj, 2011. **343**: p. d6884.
64. London, A.J., D.A. Borasky, Jr., and A. Bhan, *Improving ethical review of research involving incentives for health promotion.* PLoS Med, 2012. **9**(3): p. e1001193.
65. Thirumurthy, H., et al., *Effect of providing conditional economic compensation on uptake of voluntary medical male circumcision in Kenya: a randomized clinical trial.* Jama, 2014. **312**(7): p. 703-11.
66. Thornton, R., *The Demand for, and Impact of, Learning HIV Status.* American Economic Review, 2008. **98**(5): p. 1829-1863.
