# Supplementary material for: Financial incentives and deposit contracts to promote HIV retesting in Uganda: A randomized trial
Source: PLoS Med. 2021 May 4;18(5):e1003630. doi: 10.1371/journal.pmed.1003630 (PMC8131095; doi:10.1371/journal.pmed.1003630)
Supplement: S1 Table — (DOCX) [file pmed.1003630.s003.docx]

**S1 Table.** Instrumental variable regression results to estimate causal effect of making a deposit on HIV retesting

|  | (1) | (2) | (3) |  | (4) | (5) |
| --- | --- | --- | --- | --- | --- | --- |
| Outcome | Made baseline deposit | Retesting at 3 months | Retesting  at 3 & 6 months |  | Retesting at 3 months | Retesting  at 3 & 6 months |
| Model | OLS | OLS | OLS |  | IV | IV |
|  |  |  |  |  |  |  |
| Deposit contract group | 0.14*** |  |  |  |  |  |
|  | (0.03) |  |  |  |  |  |
| Made deposit at baseline |  | 0.47*** | 0.31*** |  | 0.21 | -0.15 |
|  |  | (0.10) | (0.08) |  | (0.37) | (0.30) |
| Constant | 0.00 | 0.36*** | 0.15*** |  | 0.38*** | 0.18*** |
|  | (0.02) | (0.03) | (0.02) |  | (0.04) | (0.03) |
|  |  |  |  |  |  |  |
| Observations | 352 | 352 | 352 |  | 352 | 352 |
| R-squared | 0.08 | 0.06 | 0.04 |  | 0.04 |  |
| *Notes:* Columns 1-3 report results of ordinary least squares (OLS) regression models. Columns 4-5 report results from instrumental variables (IV) regression models in which participants’ assignment to deposit contract group is an instrumental variable for whether a participant made a deposit at baseline. Models include observations from participants in control group and deposit contract group only. Standard errors in parentheses. | | | | | | |
| *** p<0.01, ** p<0.05, * p<0.1 | | | | | | |
