# Supplementary material for: Financial incentives and deposit contracts to promote HIV retesting in Uganda: A randomized trial
Source: PLoS Med. 2021 May 4;18(5):e1003630. doi: 10.1371/journal.pmed.1003630 (PMC8131095; doi:10.1371/journal.pmed.1003630)
Supplement: Attachment — Submitted filename: chamie.pdf [file pmed.1003630.s004.pdf]

This paper reports a trial of financial incentives for re–testing for HIV in Uganda.

I was asked for a statistical report and I interpret that to include all aspects of the design and conduct of the study.

Points of detail

Page 10 Is it possible to put 20000 UGX into some sort of context? Given the recruitment sites in Table 1 how much does a beer cost or a taxi ride?

Page 13 Page 9 tells us that the randomisation was 1:1:1 in blocks of size 9 and stratified by sex. In that case when recruitment stopped the most extreme imbalance would be 3 people. Even if both sexes stopped with that imbalance the maximum would be 6. So how do we arrive at 8 more people in one arm than another?

Page 14 Perhaps a footnote telling us what *bora bora* is? I know we can look it up on the web.

Page 14 and 15, Table 1 It is illogical to test for baseline differences. The null hypothesis is that they were drawn from the same population and any differences are due to chance. But you know for sure that they are due to chance, you randomised them (Senn, 1994). If the authors believe that something has gone wrong with the randomisation then the title of this article needs to start 'A non–randomised trial'.

Page 19 I have not carried out detailed calculations but it looks as though some of these models would predict outcomes outside [0,1] which suggests a model for odds might be more appropriate.

Point of more substance

The authors have a focus on presenting results per group without any direct comparison between groups. I suggest that presenting the risk difference with a confidence interval would be appropriate if the authors are later going to model proportions directly. That would also enable presenting number needed to treat with its confidence interval (Altman, 1998). Risk ratios or odds ratios would of course be a good option if a multiplicative scale would be preferred. Whatever course of action is taken the relevant statistics should go into the abstract as well.

Summary

The randomisation issue is a concern and the analysis does not do justice to the data.

Michael Dewey

References

D G Altman. Confidence intervals for the number needed to treat. *British Medical Journal*, 317:1309–1312, 1998.

S Senn. Testing for baseline imbalance in clinical trials. *Statistics in Medicine*, 13:1715–1726, 1994.
