# Supplementary material for: Financial incentives and deposit contracts to promote HIV retesting in Uganda: A randomized trial
Source: PLoS Med. 2021 May 4;18(5):e1003630. doi: 10.1371/journal.pmed.1003630 (PMC8131095; doi:10.1371/journal.pmed.1003630)
Supplement: Attachment — Submitted filename: Response to Reviewers PlosMed IBIS 21Mar2021.docx [file pmed.1003630.s005.docx]

**Response to Editor and Reviewer Comments**

**Manuscript Title**: A Randomized Trial of Financial Incentives and Deposit Contracts to Promote HIV Retesting

**Reference Number**: PMEDICINE-D-21-00078R1

Requests from the editors:

*Please adapt your title so that the study descriptor ("a randomized trial" falls after a colon); and include the country name.*

**Response:** We have edited our title as follows: “Financial Incentives and Deposit Contracts to Promote HIV Retesting in Uganda: a randomized trial”

*In the abstract and results section, please quote effect sizes and 95% CI for the primary endpoint findings.*

**Response**: We now quote effect sizes and 95% CI for the primary endpoint findings in the abstract and the results sections.

*Please quote study dates in your abstract.*

**Response**: We now provide study dates in our abstract.

*Please add a few final sentence to the "Methods and findings" subsection of your abstract, beginning "Study limitations include ..." or similar and quoting 2-3 of the study's main limitations.*

**Response**: We now include study limitations in the final part of the “Methods and findings” subsection of our abstract.

*After the abstract, we will need to ask you to add a new and accessible "Author summary" section in non-identical prose. You may find it helpful to consult one or two recent research papers published in PLOS Medicine to get a sense of the preferred style.*

**Response**: We now include a new and accessible “Author summary” section using non-identical prose, following our abstract.

*Please refer to figure 2 as the "Participant flowchart" or similar, rather than "CONSORT diagram".*

**Response**: We now refer to Figure 2 as the “Participant flowchart” rather than “CONSORT diagram.”

*We believe that CONSORT discourages statistical tests at baseline in randomized trials, and ask that you remove these from table 1.*

**Response**: We have removed the p-value column from Table 1.

*At line 261 and any other instances in the paper, please avoid "nearly three-fold", instead quoting actual numbers.*

**Response**: We now quote actual numbers rather than the term, “nearly three-fold,” in the first sentence of the Discussion section, as follows: “In this randomized controlled trial of financial incentives and deposit contracts to promote routine retesting for HIV among adults at increased risk of HIV in Uganda, providing financial incentives led to a 2.9-fold (52% vs. 18%) increase in retesting compared to the control of counseling on HIV retesting.”

*Throughout the text, please style reference call-outs as follows: "... HIV transmission [1,2]. Similarly ... " (noting the absence of spaces within the square brackets).*

**Response**: We have updated the citation formatting to use square brackets and to remove spaces within the brackets.

*Please remove the information on study funding from the end of the main text. In the event of publication, this information will appear in the article metadata, via entries in the submission form.*

**Response**: We have removed the information on study funding from the Acknowledgements section (at the end of the main text).

*In the reference list, please ensure that all references have full access details, e.g., reference 15.*

**Response**: We have updated reference 15 and ensured all references have full access details.

*Please ensure that journal names are abbreviated consistently.*

**Response**: We have reviewed journal name abbreviations for consistency.

*Please add a completed CONSORT checklist, labelled "S1_CONSORT_Checklist" or similar and referred to as such in the Methods section. In the checklist, please refer to individual items by section (e.g., "Methods") and paragraph number rather than by page or line numbers, as the latter generally change in the event of publication.*

**Response**: We have completed a CONSORT checklist, labelled “S1 CONSORT Checklist” and refer to it in the Methods section. Individual items are referred to by section and paragraph number, as requested.

*Please include the study protocol as a supplementary document, referred to in your Methods section, unless this is published.*

**Response**: We now include our study protocol as a supplementary document (Supplementary Materials 2, “**S2**: Study Protocol”) and refer to it in our Methods section.

*Comments from the reviewers:*

***** Reviewer #1:**

*Michael Dewey*

*This paper reports a trial of financial incentives for re–testing for HIV in Uganda.*

*I was asked for a statistical report and I interpret that to include all aspects of the design and conduct of the study.*

*Points of detail*

*Page 10 Is it possible to put 20000 UGX into some sort of context? Given the recruitment sites in Table 1 how much does a beer cost or a taxi ride?*

**Response**: We now provide greater context for the incentive amount (25,000 USh) and the deposit amount (20,000 USh) of the deposit contract in 2018, as suggested by the reviewer. Please see Methods section, page 11, paragraph 4, line numbers: 181-183.

*Page 13 Page 9 tells us that the randomisation was 1:1:1 in blocks of size 9 and stratified by sex. In that case when recruitment stopped the most extreme imbalance would be 3 people. Even if both sexes stopped with that imbalance the maximum would be 6. So how do we arrive at 8 more people in one arm than another?*

**Response**: We now further clarify our randomization procedures. Randomization cards with “scratch off” windows (so to keep staff and participants blinded to underlying randomization card distribution) were presented by study staff to each participant as a group of 9 cards, following the block order. Participants would then select one card at random and remove the “scratch off” window to reveal group assignment. Study staff then would replace the card chosen with the next randomization card in the allocation sequence (to which they were blinded). This procedure maintained blinding of study staff (i.e. they could not deduce which study arm from remaining cards in a block of 9), and also allowed participants to select their own randomization card (at random), so as to avoid the perception that study staff selected the study arm for participants. However, this process of replacing the chosen card with the first card from the next block meant there were minor differences in the number of participants in each study arm. We now clarify this process in Methods, page 11, paragraph 4, lines 174-178, as follows, “…randomized (1:1:1, by block randomization, stratified by sex, with block size=9 and allocation sequence computer-generated prior to trial initiation; *participants then selected a randomization “scratch off” card from 9 nine cards presented by staff, with replacement of each card taken with another card from the next block*) to three groups: financial incentives, deposit contracts, or control.”.

*Page 14 Perhaps a footnote telling us what bora bora is? I know we can look it up on the web.*

**Response**: We now add a footnote in Table 1 explaining what a “*boda boda*” is: this is a local term that refers to a motorcycle taxi (see pages 16-17).

*Page 14 and 15, Table 1 It is illogical to test for baseline differences. The null hypothesis is that they were drawn from the same population and any differences are due to chance. But you know for sure that they are due to chance, you randomised them (Senn, 1994). If the authors believe that something has gone wrong with the randomisation then the title of this article needs to start ‘A non–randomised trial’.*

**Response**: We have now removed the column indicating p-values testing for baseline differences from Table 1 (see pages 16-17).

*Page 19 I have not carried out detailed calculations but it looks as though some of these models would predict outcomes outside [0,1] which suggests a model for odds might be more appropriate.*

**Response**: A common form of instrumental variables analysis is the two-stage least squares (2SLS) regression model in which the first stage is an ordinary linear regression in which the outcome is the endogenous variable (choice to make a deposit) on the instrumental variable (study arm assignment). In econometrics it is standard practice to use 2SLS even for dichotomous outcomes, and there has been prior work indicating that this method is less biased that other methods such as IV-GMM and yields satisfactory confidence intervals (for example see Halpern et al. NEJM 2015, [Baicker et al. NEJM 2013](https://www.nejm.org/doi/full/10.1056/nejmsa1212321); Finkelstein et al. *Quarterly Journal of Economics* 2012). We also cite a more general paper of the IV method in the revised version: Angrist JD, Imbens GW, Rubin DB. Identification of causal effects using instrumental variables. J Am Stat Assoc 1996;91:444-455).

*Point of more substance*

*The authors have a focus on presenting results per group without any direct comparison between groups. I suggest that presenting the risk difference with a confidence interval would be appropriate if the authors are later going to model proportions directly. That would also enable presenting number needed to treat with its confidence interval (Altman, 1998). Risk ratios or odds ratios would of course be a good option if a multiplicative scale would be preferred. Whatever course of action is taken the relevant statistics should go into the abstract as well.*

**Response**: We now present direct comparisons of the primary outcome (HIV retesting at 3 and 6 months) between groups as odds ratios with 95% confidence intervals in both the Results section (see page 17-18, lines 272-281), and in the Abstract (lines 47-50).

*Summary*

*The randomisation issue is a concern and the analysis does not do justice to the data.*

**Response**: We thank Reviewer 1 for the comments and have addressed the randomization issue raised and the analysis suggestion.

***** Reviewer #2:**

*This is an interesting, well conducted and well reported study.*

*My main comment is about the fact that the take-up for the deposit contract appeared to be quite low. I was not necessarily surprised by this finding given the low average income in the study setting and therefore the predictably low ability to save. I am surprised that this did not appear as a major constraint for the feasibility of study. Specifically, I would have liked the authors to compare their finding in their pilot study (Chamie G, Ndyabakira A, Marson KG, Emperador DM, Kamya MR, Havlir DV, et al. A pilot randomized trial of incentive strategies to promote HIV retesting in rural Uganda. PLoS ONE. 2020;15(5):e0233600.) with their findings in the present study. Did they manage to get higher uptake of the deposit contracts in the pilot study? Apparently yes, since in the pilot study 93% made deposits, but only 14% in the current study.  Why and what might explain the differences in uptake between the pilot study and current study? This seems to be a key point to discuss.*

**Response**: As the Reviewer notes, we did observe higher uptake of the deposit contracts in the pilot study referenced (also conducted by our study group: Chamie et al, PLoS One, 2020). We attribute the substantially higher participation in the pilot study deposit contract group to the *much lower amount* requested for the deposit. In the pilot study, the deposit amounts of the two deposit arms were equal to or less than the financial incentive provided for baseline testing (pre-enrollment and randomization) at the study clinic. In this present trial, as noted in our discussion (see Discussion, page 24, lines 381-384), “*we increased the deposit amount in order to require a larger pre-commitment of one’s own money and thus generate a greater sense of loss aversion. However, perhaps as a consequence, we observed relatively low baseline deposit contract uptake (14%).*” We agree this is a key point for discussion, and now add further reflection on what may have happened had we lowered the deposit amount in the deposit contract group (see Discussion, pages 24-25, lines 384-386, “*We suspect that had our deposit amount been lower, we would have observed higher baseline deposit contract uptake, but possibly also lower testing uptake among those making deposits.*” We also conclude this paragraph with the following statement (lines 392-394), *“Future research could consider comparing differing deposit contract amounts and interest earned to increase participation while maintaining the potential of leveraging precommitment and loss aversion for behavior change.”*

*Given the low uptake for the deposit contracts in the current study, very little can be concluded about the comparison between cash incentives and deposit contracts, which, I suppose, was the main objective of the study. The remaining result is about the effectiveness of the cash incentives, but that effectiveness has already been established (see Lee R, Cui RR, Muessig KE, Thirumurthy H, Tucker JD. Incentivizing HIV/STI Testing: A Systematic Review of the Literature. AIDS Behav. 2013.)*

**Response**: The objective of the study was to compare the effectiveness of offering deposit contracts to the comparator arms of offering financial incentives or control (current standard of care, which is counseling people to retest). We believe it is important to have evidence on the overall effectiveness of deposit contracts. For example, if those who make a deposit are very likely to retest and the deposit uptake is sufficiently high, it may still be more effective overall than incentivizing everyone (our results show this does not appear to be the case). Furthermore, we also conduct instrumental variables analysis to assess the causal effect of choosing to make a deposit. In addition, although we appreciate that financial incentives have been shown to be effective in increasing uptake of one-time HIV testing, their effectiveness in promoting routine retesting (i.e. more than one occasion over time, such as at the 3- and 6-month time points evaluated in our primary endpoint) has not been established. Indeed, this is the driving motivation in our trial for studying a repeated health behavior, HIV retesting, that is necessary to increase HIV status awareness (a major global health priority) and that requires sustained behavior change.

*Minor comment:*

*I am surprised not to have found one of the first study testing incentives for HIV testing in the reference list:*

*Thornton RL. The Demand for, and Impact of, Learning HIV Status. Am Econ Rev. 2008;98(5):1829-63.*

**Response**: In our initial submission we had relied on the systematic review by Lee et al, to provide a single reference for the evidence base supporting incentives for promotion of one-time HIV testing, rather than referencing the studies that support this finding. However, we agree with the reviewer that this seminal study by Thornton et al, Am Econ Rev from 2008 is among the first studies establishing the effectiveness of incentives for HIV testing, and we now include this study in our references (see Introduction, page 8, line 118, citation 17, and References).

***** Reviewer #3:**

*A Randomized Trial of Financial Incentives and Deposit Contracts to Promote HIV Retesting*

*Manuscript Number:  PMEDICINE-D-21-00078R1*

*This manuscript reports the results of a three-group randomized, controlled trial to determine the effectiveness of 1) financial incentives and 2) deposit contracts vs. 3) control in achieving HIV re-testing at both 3 and 6 months post-randomization among people at high risk for contracting HIV in southwestern Uganda. Eligible individuals were those who presented for an evaluation and who were 18-59 years of age, tested negative for HIV, and reported at least one of the following risk factors in the prior 12 months: 1) >1 partner; 2) a known HIV-infected partner; 3) a history of a sexually transmitted infection; or 4) paid or received money or gifts in exchange for sex. Self-report (at screening/baseline) and HIV testing data (screening/baseline and follow-up) were collected over a ~6-month observation period.*

*Strengths of this study are its large sample size, three-arm randomized and controlled design, rapid recruitment period, and high-risk target population. The paper is well organized, clearly written and will add to the body of literature on the effectiveness of financial incentives and deposit contracts. This reviewer found no major issues and only a handful of minor issues (described below) that authors may consider addressing.*

**Response**: We are pleased that the Reviewer finds the paper well organized, clearly written and an addition to the body of literature on financial incentives and deposit contracts.

*Major Issues:  None*

*Minor Issues:*

*1)	Lines 134-135:  Consider including a brief description of measures assessed via the baseline questionnaire. If not feasible due to space limitations, consider referring readers to Table 1 for a list of the measures. Also, consider clarifying whether any follow-up questionnaire was administered at 3- and 6-month follow-ups.*

**Response**: We now include a brief description of the types of measures assessed via the baseline questionnaire (see Methods section, page 11, lines 173-174). We also now clarify that a brief follow-up questionnaire was administered to participants who retested at 3- and 6-months follow-up (see Methods section, page 12, lines 203-205).

*2)	Line 147:  Figure 1 refers to "cash transfer", yet this term is not addressed within the text. Does this payment correspond to the "one-time reimbursement" of $2.70 paid to individuals who completed the screening/baseline evaluation (as mentioned in line 118)?*

*a)	If yes, then I recommend using parallel terminology and $ amount in the text and figure to add clarity.*

*b)	If no, then I recommend the term, "cash transfer", be briefly defined or described within the text prior to being used in the figure.*

**Response**: Yes, the “cash transfer” in Figure 1 does correspond to the “one-time reimbursement” paid to individuals who completed the screening. We now use parallel terminology and dollar/shilling amounts in the text and figure for clarity. The text now reads (lines 155-156), “The cards indicated that those who came for an evaluation would receive a one-time cash transfer of 10,000 Ugandan Shillings (USh) (US$2.70 in 2018) for reimbursement of travel expenses to reach the clinic.” Figure 1 now indicates $2.70 in reference to the amount of the cash transfer (instead of $3).

*3)	Line 188:  The recruitment period is clearly describing. For clarity and context, consider clearly stating the full study observation period and/or "stop date" (last date on which data were collected for last randomized participant).*

**Response**: We now include the stop date for trial participants in the Results section, as follows: “Trial activities for the final participants were completed on August 7, 2019.” (see page 21, lines 314-315).

*4)	Lines 265-266:  The phrase, "Further cost-effectiveness modeling…" sounds like some cost-effectiveness modeling may already have been performed and additional modeling is recommended. Is this the case?*

*a)	If yes, please briefly present results of any cost-effectiveness modeling already performed.*

*b)	If no, please eliminate "Further" and start the sentence with "Cost-effectiveness modeling…" to avoid confusion.*

**Response**: No, we have not conducted any cost-effectiveness modeling, and we now eliminated “Further” from the start of this sentence to avoid confusion (see page 22, line 325).

*5)	Line 332:  Consider addressing the short observation period (only 6 months) as a study limitation, particularly because lines 66-67 indicate that Uganda Ministry of Health recommends HIV retesting every 3 months for "key populations" which (presumably) includes the high-risk individuals recruited for this study.*

**Response**: We now include the 6-month observation period of the trial as a study limitation. Though this trial period does include two opportunities (at 3 and 6 months) for HIV retesting of persons at increased risk of HIV, a longer trial period may have captured longer term trends in intervention effectiveness and retesting behavior. We now note in our Discussion section, page 25, lines 403-404: “Lastly, we did not evaluate retesting beyond the 6-month trial period or post-trial retesting behavior.”

*****
