# Supplementary material for: Financial incentives and deposit contracts to promote HIV retesting in Uganda: A randomized trial
Source: PLoS Med. 2021 May 4;18(5):e1003630. doi: 10.1371/journal.pmed.1003630 (PMC8131095; doi:10.1371/journal.pmed.1003630)
Supplement: Attachment — Submitted filename: Response to PLoS Med Editors v12Apr2021.docx [file pmed.1003630.s006.docx]

**Response to Editor and Reviewer Comments (R2)**

**Title**: “Financial Incentives and Deposit Contracts to Promote HIV Retesting in Uganda: a randomized trial.”

**Reference** **Number**: PMEDICINE-D-21-00078R2

**Requests from Editors:**

*1) Please finalize the arrangements for data deposition and release.*

**Response**: We have arranged for data deposition into the Harvard dataverse site (<https://dataverse.harvard.edu>), along with the data dictionary. The data can be accessed at the following url: <https://dataverse.harvard.edu/dataset.xhtml?persistentId=doi:10.7910/DVN/PXCL0W>

*2) Please add a sentence, say, at line 51 to quote the number, and distribution by study arm, of seroconversions.*

**Response**: We have added a sentence at line 52 that provides the number and distribution by study arm of seroconversions, as follows: “*Among 282 participants who retested for HIV, three (1%; 95%CI: 0.2-3%) seroconverted: one in the incentive group and two in the control group.*”

*3) Throughout the text, please move reference call-outs before punctuation (e.g., " ... HIV transmission [1,2].").*

**Response**: We have moved all reference call outs before punctuation throughout the text in the revised manuscript.

*4) In the reference list, please abbreviate journal names consistently (e.g., "PLoS Med.").*

**Response**: We have reviewed and edited our reference list for consistency of abbreviated journal names.

Comments from Reviewers:

****Reviewer #1:

The authors have addressed my points and have clearer up the point about the randomisation.

Michael Dewey

*** Reviewer #2:

Thank you for your responses to my comments.
I remain convinced of the relevance of my second comment:
"Given the low uptake for the deposit contracts in the current study, very little can be concluded
about the comparison between cash incentives and deposit contracts, which, I suppose, was the
main objective of the study. The remaining result is about the effectiveness of the cash
incentives, but that effectiveness has already been established (see Lee R, Cui RR, Muessig KE,
Thirumurthy H, Tucker JD. Incentivizing HIV/STI Testing: A Systematic Review of the Literature.
AIDS Behav. 2013.) "
Your answer is technically correct but does not convince me that the results from this randomized control trial are sufficiently novel and important to be published in PLoS Medicine.

*** Reviewer #3:

The "minor issues" that I raised were satisfactorily addressed in this revision.

****

**Response**: We again thank the reviewers for their thoughtful comments.
